# Detection of statin-induced rhabdomyolysis and muscular related adverse events through data mining technique

**DOI:** 10.1186/s12911-022-01978-4

**Published:** 2022-09-05

**Authors:** Patratorn Kunakorntham, Oraluck Pattanaprateep, Charungthai Dejthevaporn, Ratchainant Thammasudjarit, Ammarin Thakkinstian

**Affiliations:** 1 Department of Clinical Epidemiology and Biostatistics, Faculty of Medicine Ramathibodi Hospital, Mahidol University, 270 Rama VI Rd, Ratchathewi, Bangkok, 10400 Thailand; 2 Department of Medicine, Faculty of Medicine Ramathibodi Hospital, Mahidol University, 270 Rama VI Rd, Ratchathewi, Bangkok, 10400 Thailand

**Keywords:** Statin, Rhabdomyolysis, Drug interaction, Data mining, Bayesian network, Random forests, Extreme gradient boosting

## Abstract

**Background and objective:**

Rhabdomyolysis (RM) is a life-threatening adverse drug reaction in which statins are the one commonly related to RM. The study aimed to explore the association between statin used and RM or other muscular related adverse events. In addition, drug interaction with statins were also assessed.

**Methods:**

All extracted prescriptions were grouped as lipophilic and hydrophilic statins. RM outcome was identified by electronically screening and later ascertaining by chart review. The study proposed 4 models, i.e., logistic regression (LR), Bayesian network (BN), random forests (RF), and extreme gradient boosting (XGBoost). Features were selected using multiple processes, i.e., bootstrapping, expert opinions, and univariate analysis.

**Results:**

A total of 939 patients who used statins were identified consisting 15, 9, and 19 per 10,000 persons for overall outcome prevalence, using statin alone, and co-administrations, respectively. Common statins were simvastatin, atorvastatin, and rosuvastatin. The proposed models had high sensitivity, i.e., 0.85, 0.90, 0.95 and 0.95 for LR, BN, RF, and XGBoost, respectively. The area under the receiver operating characteristic was significantly higher in LR than BN, i.e., 0.80 (0.79, 0.81) and 0.73 (0.72, 0.74), but a little lower than the RF [0.817 (95% CI 0.811, 0.824)] and XGBoost [0.819 (95% CI 0.812, 0.825)]. The LR model indicated that a combination of high-dose lipophilic statin, clarithromycin, and antifungals was 16.22 (1.78, 148.23) times higher odds of RM than taking high-dose lipophilic statin alone.

**Conclusions:**

The study suggested that statin uses may have drug interactions with others including clarithromycin and antifungal drugs in inducing RM. A prospective evaluation of the model should be further assessed with well planned data monitoring. Applying LR in hospital system might be useful in warning drug interaction during prescribing.

**Supplementary Information:**

The online version contains supplementary material available at 10.1186/s12911-022-01978-4.

## Introduction

Rhabdomyolysis (RM) is a life-threatening adverse drug reaction (ADR) which commonly presents with myopathy, myalgia, and dark urine, together with or without the rising of creatine kinase (CK) [1–3]. The most severe complication, acute kidney injury (AKI), was found in about 10–55% of RM patients, which resulted in multiple organs failure and deaths about 7.1–13.0% per year [3, 4]. Treatment of RM is a supportive care with intravenous fluid therapy [1]. The estimated cost per quality-adjusted life years (QALY) to treat RM was 69,742.5 USD per year [5], and the treatment cost even raised to 11,000 USD per episode of AKI [6].

Medication use was reported as one of the major causes of RM. The most common drugs are antihyperlipidemic agents such as simvastatin, atorvastatin, and rosuvastatin, regardless of co-administration with other drugs with the rate of RM ranged from 0.5 to 3.7, and as high as 18.6 to 22.5 per 10,000 person-years for combined therapy [7, 8]. Lipophilicity (e.g., atorvastatin, fluvastatin, pitavastatin, and simvastatin) have higher risk for induction of RM compared to hydrophilic (e.g., pravastatin and rosuvastatin) group [9]. In addition, statin also interacts with other drugs including gemfibrozil, amiodarone, digoxin, cyclosporine, colchicine, diltiazem, and verapamil. Nonetheless, the early detection of ADRs could reduce the treatment costs around 1,400 USD per patient [10].

Data mining techniques (e.g., disproportional analysis (DPA) and machine learning-based model (ML)) have been applied to early detect ADRs. The DPA is a fast and inexpensive method, which is widely used for signal detection in spontaneous reporting systems (SRSs) [11–15], but it is not appropriate for data with high correlation among predictors, or a large dimensional data in electronic health records (EHRs). Data mining from SRS plus EHR could improve the accuracy of signal detection compared to using the information of SRS alone [16]. The ML algorithms have been proposed to solve those limitations of SRSs [17, 18]. Nowadays, there is no consensus on which method is the most appropriate to assess the drug-ADR associations [11, 19].

A previous study [18] proposed a text mining algorithm for detection of statins induced myopathy or RM from EHRs of a hospital in Singapore. The algorithm had high precision and recall, but the algorithm required discharge summaries in English. Hence, it could not apply to hospitals in Thailand, in which discharge summaries were handwritten with various abbreviations and mixed languages. In addition, their black box algorithms could not explain which drugs increased the risk of RM. Therefore, the study had two research questions as follows: First, did statin alone associate with RM or muscular related ADR occurrence? Second, was statin effect on RM or muscular related ADR modified by other drugs? The study was conducted which aimed to explore the association between use of statin alone or in combination with other drugs and RM or muscular related ADR using the EHRs of Ramathibodi Hospital.

## Methods

### Data source

Five EHRs of Ramathibodi Hospital including patient registration, medication, laboratory, diagnosis/procedure, and SRS databases were retrieved from the hospital informatics system during 1st January 2012 to 31st December 2019. Patients were identified and included into the study if they were aged 18 years or older and had been prescribed statins as details in Additional file 1: Table A.1. Statins were categorised into 2 groups, i.e., hydrophilic (pravastatin and rosuvastatin) and lipophilic (atorvastatin, fluvastatin, pitavastatin, and simvastatin) statins. Other features of those eligible patients, as shown in Additional file 1: Table A.1, were also retrieved. Standard codes were used for information extractions including Thai Medicines Terminology (TMT), Thai Medical Laboratory Terminology (TMLT), International Statistical Classification of Diseases and Related Health Problems 10th Revision Thai Modification 2010 (ICD-10-TM), and International Classification of Diseases 9th Revision Clinical Modification (ICD-9-CM), and the hospital codes, see Additional file 1: Table A.2–A.4. The study had been approved by the ethics committee of the Faculty of Medicine Ramathibodi Hospital, Mahidol University. All identified data were encrypted using the MD5 hash function.

### Outcome of interest

**Fig. 1:**
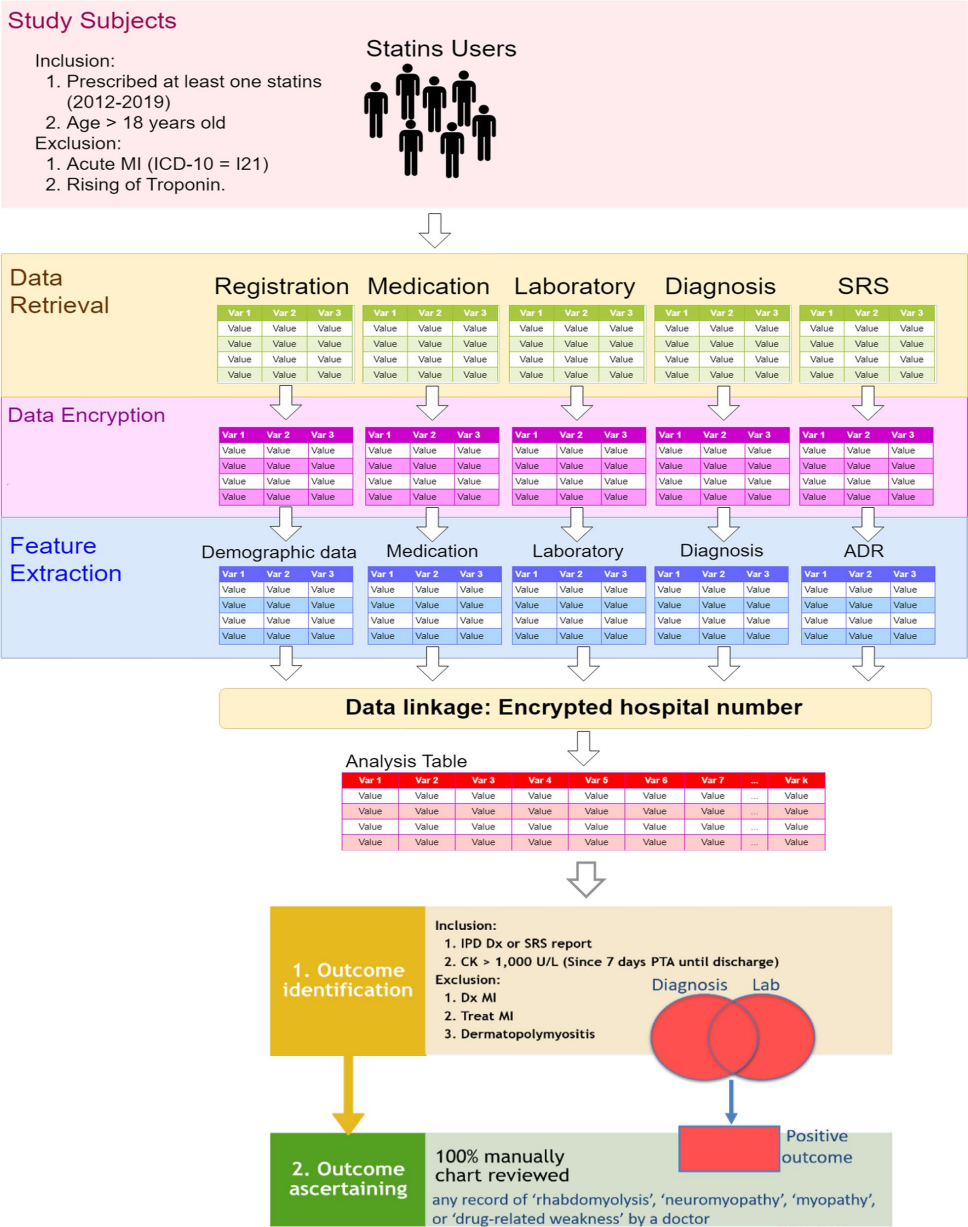
Steps for identification and ascertainment of the outcome

An occurrence of RM or muscular related adverse event, hereafter called ‘RM’, was the outcome of interest. Two steps were performed as described by Fig. 1. First, RM was initially identified in a cohort of patients with statin users by linking a cohort database with ICD10-CM (see codes in Additional file 1: Table A.4) and creatine kinase (CK) level of > 1000 U/L from laboratory databases. RM patients with myocardial infarction (MI), dermatomyositis, or polymyositis were excluded. Second, identified RMs were ascertained by manually review of EMRs by our research team which consisted of a pharmacist and four physicians. The reviewers checked whether there was any report of ‘rhabdomyolysis’, ‘neuromyopathy’, ‘myopathy’, or ‘drug-related weakness’ in the hospital’s EHR to confirm as the RM outcome.

### Patient’s timeline

The individual patient’s timeline was constructed based on the use of hydrophilic and lipophilic statins regardless of switching statins. An episode of each person-time started at the date of the first prescription of the statin groups and ended with the RM occurrence. The duration of taking statins was calculated from the drug regimen of each prescription. If a patient had multiple episodes of RM, the beginning of the next episode would be on the date of prescribing statins after the end of the previous episode.

### Data preparation

The underlying diseases and demographic data were included as baseline characteristics for individual person-time. Other extracted features were mapped to each event with different criteria depending on the type of outcome, clinical knowledge, and real practice as summarised in Additional file 1: Table A.5. Then the dataset was split into train and test datasets with a ratio of 80:20 for development and validation of the model, respectively.

A total of 122 categorical and continuous features were considered to include in analysis, see Additional file 1: Table A.6. The feature of interest was statin use considered as statin only or combined with other lipid lowering drugs, type of statin (i.e., lipophilic and hydrophilic coded as 1 and 0, respectively), and high or low/standard dose of each statin. In addition, 77 medications that could increase statin levels were also considered. Furthermore, 26 medications known in the literatures as inducing RM occurrence were also considered. Finally, other confounding features were also considered including 2 demographic data, 8 laboratory tests, 5 underlying diseases, and any of 16 comorbidities. These binary features were coded as 1 for presence and 0 otherwise. Age was categorised according to a median of 65 years. Statin dose was calculated based on a number of doses taken per day, then compared to the number of defined daily doses (DDD) recommended by the World Health Organisation (WHO) [20]. The average number of DDD during a period of the event was classified into high dose if DDD was higher than 1, otherwise as low (standard) dose. Other drugs were assumed to be taken as 1 DDD, and periods of treatment were calculated. If a period for taking a drug was overlapped with the period of event occurrence, it was identified as co-administration with code of 1, and 0 otherwise.

Laboratory values measured at/close to statin prescriptions were used for analysis. Those laboratory features were classified into binary features coded as 0 and 1 for normal abnormal based on the normal reference ranges [21, 22]. The level of eGFR was classified as declining kidney function if eGFR < 60 mL/min/1.73 m^2^ (code 1), otherwise it was normal (code 0). For tests of lipid profile, only TG and LDL were included because doctors considered to prescribe any regimen of statins based on these laboratory results.

Eight lab data were missing, which ranged from 0.004 to 0.08%. Those data were imputed using multiple imputations by chained equations (MICE) of linear regression with 10 imputations [23], see Additional file 1: Table A.8, as follows: First, Scr, the smallest missing value, was initially imputed based on 114 features. Then, the imputed Scr plus other 114 features were used to predict the next second least missing AST. Next, the Na was predicted based on imputed Scr and imputed AST plus 114 completed features. The process was repeated until the last feature, i.e., TG. The whole process was repeated 10 times to get 10 imputed data. Distributions of those imputed features are described, see Additional file 1: Table A.8

### Feature selection

Feature selection was performed as follows. First, features were considered based on biological mechanisms in relationships with RMs. Second, univariate analysis was applied using Pearson’s Chi-squared test or Fisher’s exact test where appropriate [24] to assess association between each feature and RM. Third, a 1000-replication bootstrapping with multiple logistic regression was applied by simultaneously fitting all features whose p-value was < 0.1 in the univariate analysis, and non-significant features by expert opinions, which were age and common drugs that may have drug interaction with statin [9, 10] including amiodarone, ciprofloxacin, digoxin, diltiazem, ezetimibe, and gemfibrozil which were reconsidered to include in the model. Lastly, multicollinearity was verified by calculating the variance inflation factor (VIF) score. [9, 10].

### Model development

To estimate the effect size of the association between drug(s) and outcome, the study has proposed 4 models, i.e., logistic regression (LR), Bayesian network (BN), random forests (RF), and extreme gradient boosting (XGBoost). Data were split into 80:20 for train and test sets using random without replacement method with consideration that patients must be included in each data set only once. All models were learnt on the same train dataset with the same selected features. Moreover, the performance of models were evaluated based on the same test dataset.

#### Logistic regression (LR)

LR model was created through STATA® version 16 by including all features suggested by univariate analysis and expert opinion. Then only the first level of statin-drug interaction was considered for analysis. Also, the problem of zero RM outcome was avoided, thus the interaction effect of a statin group was compared with the rest instead of directly fitting with the interaction term. For example, the interaction effects were considered by comparing the odds of the outcome when using high-dose lipophilic statins alone and when using other groups of statins with a concurrent drug.

#### Bayesian network (BN)

There were 2 steps of developing BN model. Firstly, structure of model was created based on clinical knowledge using GeNIe® modeler which is a free graphic user interface (GUI) software application. However, a large number of parent nodes contributed to the problem of high computation for the conditional probability table and complicated learning inference. Therefore, a parent divorcing and Noisy-OR would be applied if more than 20 features were eventually chosen in accordance with literatures [25, 26]. Secondly, model parameters were learnt on the train dataset, and the conditional and the marginal probability of child and parent nodes were calculated and directly emerged on themselves. Values of information of features were also diagnosed and ranked. The highest diagnostic value meant the most relevant features that were used to predict the outcome regardless of parent or child nodes of the outcome. Moreover, a new dataset which consisted of all possible interaction terms was simulated for analysis of the drug interactions.

#### Random forests (RF)

RF is a machine learning model which consists of a large number of individual decision trees that operate as an ensemble. Each tree in the random forest gives a class prediction, then all trees are combined. The majority votes from all trees will suggest the model prediction. The proposed model was developed using scikit-learn Python’s library, called ‘RandomForestClassifier’. In addition, the model used the “balanced” mode to augment imbalanced data by automatically adjusting weights inversely proportional to class frequencies in the dataset; which resulted in balanced data with a ratio of 1:1 for Rhabdomyolysis and non-Rhabdomyolysis groups. The model was learnt on the train dataset and hyperparameters tuned to obtain the optimal model. Tuning hyperparameters were set as follows: (1) the maximum tree depth of 3 showed an optimal parameter, (2) the maximum number of features at each split was 10, (3) the minimum number of samples required at each split was 3, (4) the number of trees in the forest was 40 and (5) The ‘random state’ was set at 0 to control the randomness of bootsrap samples. Finally, the Tree Shapley Additive Explanations (SHAP) [27–29] was applied to show feature importance and whether the feature had a positive or negative impact on predictions.

#### Extreme gradient boosting (XGBoost)

XGBoost is an ensemble method using gradient boosting technique to optimise and minimise errors of the model using xgboost Python’s library. The model was trained on the train dataset and the ‘random_state’ was set to 0. Since the data was imbalanced between the Rhabdomyolysis and non-Rhabdomyolysis groups, data was augmented and balanced by the scale_pow_weight (calculated by dividing a total number of negative instances by a total number of positive instances). Tuning hyperparameters for model optimising were set as follows: (a) learning rate was set at 0.01, (b) a maximum depth of the tree was 3, (c) number of features used to consider at each split (colsample_bytree) was 0.8, and (d) a number of gradient boosted trees was 20. Finally, SHAP algorithm was applied to explain the importance of features which contributed to the model.

### Model evaluation

All performances of LR, BN, RF, and XGBoost were evaluated based on the same test dataset. Sensitivity or recall was the most important metric for this study as the model should correctly detect the occurrence of outcome compared with the actual positive outcome. Specificity and area under the receiver operating characteristic (ROC) were also measured. Moreover, the low prevalence of outcome induced the imbalanced dataset. All models had calibrated threshold of measurements. The threshold of measurements for LR and BN were 0.002 which closed to the prevalence of the outcome. Whereas the threshold of RF and XGBoost were 0.5. Finally, the net reclassification index (NRI) [30] was estimated along with its 95% confidence interval (CI) by comparing BN, RF, and XGBoost with the LR models.

## Results

A total of 939 patients with 70,470 observations were included in a cohort of statin users. Of them, 8,791 (12.5%) and 61,679 (87.5%) used hydrophilic and lipophilic statins, see Additional file 1: Table A.7. A total of 96 patients with 104 episodes were confirmed as having RM outcome with a prevalence (95% CI) of 0.15% (0.12%, 0.18%).

Of 104 RM episodes, 56, 35, 11, 1, and 1 used simvastatin, atorvastatin, rosuvastatin, pitavastatin, and pravastatin. As depicted in Additional file 2: Fig. A.1, 89 patients had got one episode of outcome occurrence (i.e., pattern number 2 and 3), whereas only 7 patients had multiple episodes (i.e., pattern number 4) in which, 3 of them had ended with the positive outcome at every period of taking statins.

Most patients were female (63%), mean age was 67 years, and 66% had hypertension. About 64% had concurrently used statin and other drugs, in which the top 3 ranking drugs were lorazepam (19%), carvedilol (8%) and alprazolam (7%), shown in Additional file 2: Fig. A.2. Other characteristics were reported in Additional file 1: Table A.8.

### Features transformation and selection

Of 122 features, defined daily dose (DDD) and laboratory features were categorised based on clinical knowledge, and age was categorised according to a median. Distributions of features in train and test datasets were quite similar, see Additional file 1: Table A.9. Most statins users were aged 65 or older (59%) and taking low-dose statins (76%) as well as having normal laboratory results. Eventually, 18/122 features were included for model development obtained from the univariate analysis as well as expert’s judgements as listed in Additional file 1: Table A.10. Those included 11 significant features (statin, antifungals, carvedilol, clarithromycin, colchicine, cyclosporine, ticagrelor, hypertension, AST, eGFR, and LDL) and 7 expert-opinion features (i.e., age, amiodarone, ciprofloxacin, digoxin, diltiazem, ezetimibe, and gemfibrozil).

### LR findings

The odds ratio (OR) of the proposed LR was reported in Table 1. The results demonstrated that taking a high-dose lipophilic statin was about 23% higher odds of having outcome [OR = 1.227 (0.533, 2.829)] relative to taking low-dose hydrophilic statin, but this was not statistically significant. Additionally, other medications were considered and found that taking antifungals, clarithromycin, ticagrelor, cyclosporine, carvedilol, and colchicine seemed to increase odds of having outcome more than not taking those medications, but only clarithromycin was significant with the OR (95% CI) of 2.777 (1.112, 6.934). Drug interactions between those drugs and high-dose lipophilic statin were further explored indicating the odds of having outcome were significantly increased in taking high-dose lipophilic statin with clarithromycin and clarithromycin plus antifungals with the ORs (95% CI) of 5.662 (1.035, 30.962) and 16.219 (1.775, 148.226), respectively.

**Table 1:** Logistic multivariate regression results

Variables	OR	SE	z	P-value	95% CI
Intercept	0.000	0.000	− 13.160	< 0.001*	(0.000, 0.000)
*Statin groups*					
0. Low-dose hydrophilic	1.000	–	–	–	–
1. High-dose hydrophilic	0.811	0.565	− 0.300	0.763	(0.207, 3.177)
2. Low-dose lipophilic	0.743	0.306	− 0.720	0.470	(0.331, 1.664)
3. High-dose lipophilic	1.227	0.523	0.480	0.630	(0.533, 2.829)
*Medications*					
Antifungals	2.865	2.095	1.440	0.150	(0.683, 12.008)
Clarithromycin	2.777	1.297	2.190	0.029*	(1.112, 6.934)
Ticagrelor	2.220	1.344	1.320	0.188	(0.678, 7.271)
Cyclosporine	2.127	1.306	1.230	0.219	(0.638, 7.089)
Carvedilol	1.534	0.442	1.490	0.138	(0.872, 2.697)
Colchicine	1.327	0.459	0.820	0.413	(0.674, 2.613)
Amiodarone	0.654	0.319	− 0.870	0.384	(0.251, 1.703)
Digoxin	0.594	0.441	− 0.700	0.483	(0.138, 2.549)
Diltiazem	0.865	0.451	− 0.280	0.781	(0.311, 2.405)
Ezetimibe	0.712	0.256	− 0.950	0.344	(0.352, 1.439)
Ciprofloxacin	0.419	0.196	− 1.860	0.063	(0.167, 1.049)
Gemfibrozil	0.508	0.513	− 0.670	0.503	(0.070, 3.674)
*Other covariables*					
AST (> 30 U/L)	35.294	20.824	6.040	< 0.001*	(11.104, 112.181)
eGFR (< 60 mL/min/1.73 m^2^)	2.360	0.617	3.280	0.001*	(1.413, 3.940)
Hypertension	1.774	0.504	2.020	0.044*	(1.017, 3.095)
Age (> 65 years old)	0.640	0.157	− 1.820	0.069	(0.396, 1.036)
LDL (> 125 mg/dL)	0.409	0.122	− 3.010	0.003*	(0.228, 0.733)

Variables	Odds Ratio	Std. Err	z	P-value	95% CI
*Interaction effects of high-dose lipophilic statin*					
+ Clarithromycin	5.662	4.908	2.000	0.046*	(1.035, 30.962)
+ Antifungals	5.840	6.071	1.700	0.090	(0.761, 44.796)
+ Clarithromycin + Antifungals	16.219	18.309	2.470	0.014*	(1.775, 148.226)
+ Ticagrelor	4.526	4.256	1.610	0.108	(0.717, 28.584)
+ Cyclosporine	4.337	4.157	1.530	0.126	(0.662, 28.389)
+ Carvedilol	3.127	2.430	1.470	0.142	(0.681, 14.344)
+ Colchicine	2.705	2.180	1.230	0.217	(0.558, 13.125)

* *p* < 0.05

The odds of outcome were also significantly increased for rising AST, poor renal function (reduction of eGFR), and having hypertension with the ORs (95% CI) of 35.294 (11.104, 112.181), 2.360 (1.413, 3.940), and 1.774 (1.017, 3.095), respectively.

### BN findings

**Fig. 2:**
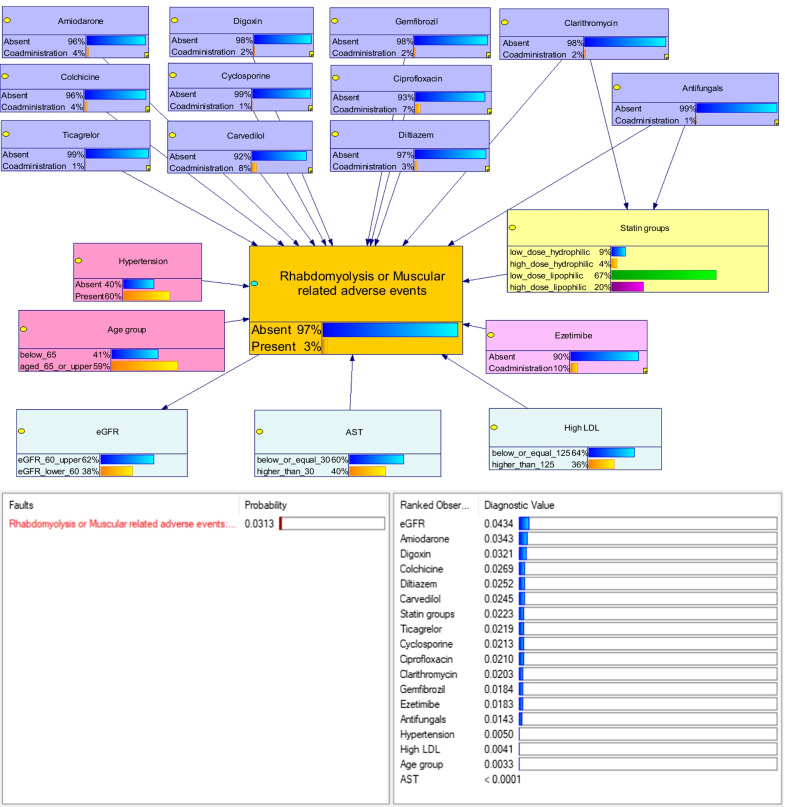
Graphical model and diagnostic results of BN. Left pane displayed the conditional probability of an outcome based on the train dataset, right pane showed ranking of the features from most to least information

Eighteen features were considered in the BN graphical model, in which eGFR was assigned as a child node, whereas the remaining played as parent nodes of the outcome, see Fig. 2. Clarithromycin and antifungals were directly associated with the RM outcome and also could be directly associated through statin users. All nodes were configured as ranked chance-general node with different diagnostic types, i.e., ‘fault’ for outcome and ‘observation’ for features. It can be distinguished that eGFR had the highest discriminatory value used to predict the outcome, whereas AST level had the least diagnostic value. The conditional probability of outcome given features (all parent nodes) was approximately 3%, whereas the conditional probabilities of the outcome given individual features were estimated, see Additional file 1: Table A.11. Taking low to high dose hydrophilic statins raised the probability of outcome from 6 to 8%, whereas low to high-dose lipophilic statins increased the probability of outcome only from 2.35 to 3.36%. Taking statins with other drugs also increased the probability of outcome, especially antifungals, ticagrelor, cyclosporine, digoxin, and clarithromycin, as shown in Table 2. For instance, occurrence of outcome increased from 6.35 to 38.30%, 40.33%, 37.89%, and 40.03% if taking low-dose hydrophilic statin with antifungals, cyclosporine, digoxin, and ticagrelor, respectively. Probability of outcome occurrence was even increased in these corresponding drug interactions with high-dose hydrophilic statin with probabilities of 41.49%, 45.05%, 41.31%, and 41.81%, respectively. Poor renal function also increased the probability of outcome occurrence of 5.64%, whereas other co-variables had only slightly changed the probability.

**Table 2:**
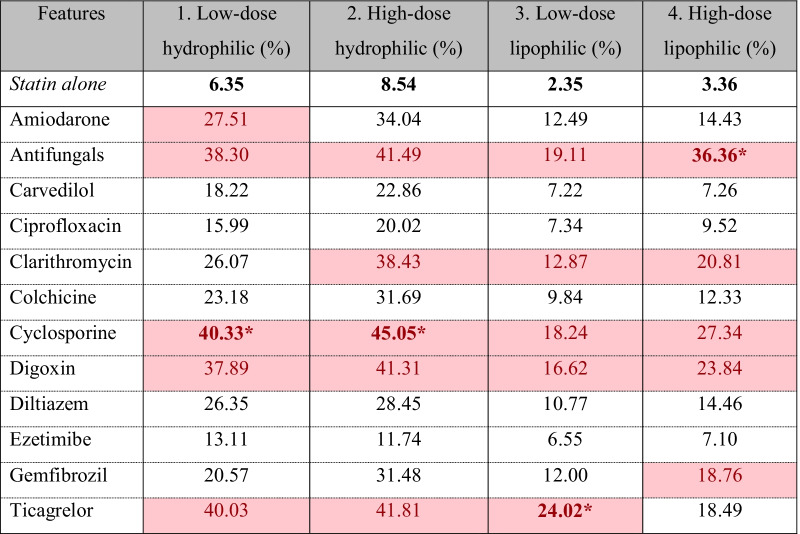
Conditional probabilities of the outcome predicted from statin-drug interaction effects

*The highest probability of outcome which was compared with other combinations within each statin group

Highlight showed the top 5 highest probabilities of the outcome for each statin group

The conditional probability of outcome occurrence with different drug combinations were estimated, see Additional file 1: Table A.12–15. According to the analysis of all possible interactions, a patient aged less than 65 who had hypertension, poor renal function, elevated AST, and concurrent use of low-dose lipophilic statins, colchicine, cyclosporine, diltiazem, and ezetimibe, had the highest chance of outcome occurrence (80%), see Additional file 1: Table A.16. Moreover, the model could predict the probability of outcome even though there was an unknown feature of individual case.

### RF findings

**Fig. 3:**
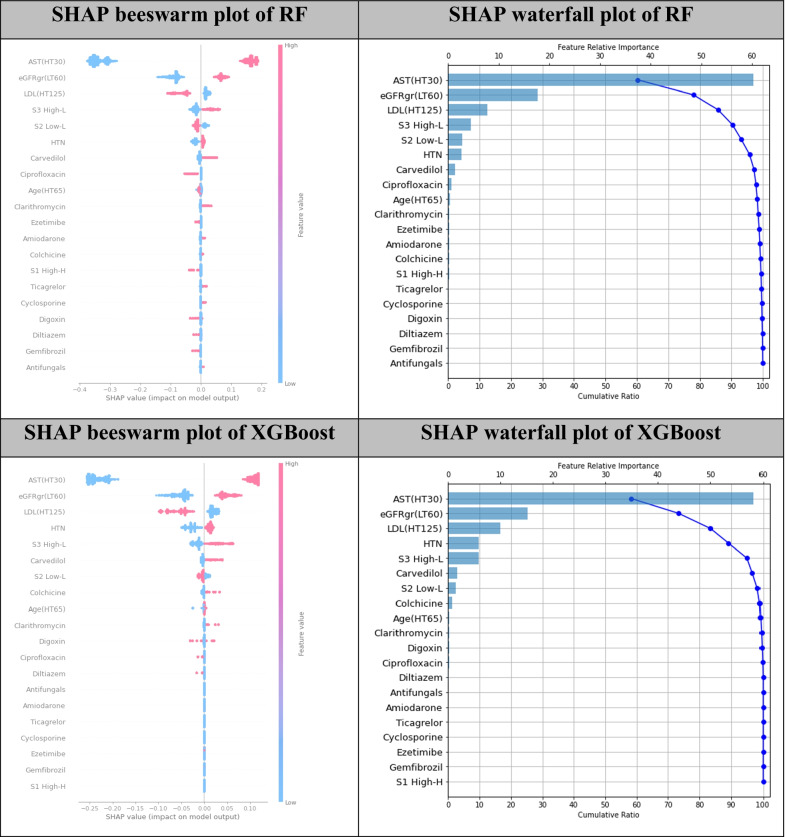
Estimation of feature important values by SHAP analysis for RF and XGBoost

As result in Fig. 3, a beeswarm plotted the SHAP value on x-axis and features which were ranked by SHAP values on the y-axis. A feature with the highest mean SHAP value was located on top of the chart shaded with red colour whereas the least SHAP value was located on the bottom with blue colour. We found that the top 5 features with high impacts on RM prediction were AST, eGFR, LDL, high-dose lipophilic statin and low-dose lipophilic statin. The chart demonstrated that patients with abnormal AST (> 30 U/L) lead to higher chance of RM occurrence, whereas patients with normal AST (≤ 30 U/L) contributed to lower RM risk. Patients with eGFR less than 60 mL/min/1.73 m^2^ were also higher risk of RM, which were similar to a patients who took high-dose lipophilic statin. Conversely, patients with abnormal LDL (> 125 mg/dL) and patients who took low-dose lipophilic statin were lower RM risks.

A waterfall plot demonstrated a combination of bar chart and line chart. The bar chart indicated feature relative importance whereas the line chart showed the cumulative ratio of SHAP values ranged from 0 to 100 percent. As suggested by the beeswarm plot, AST was the most important feature followed by eGFR, LDL, high-dose lipophilic statin, and low-dose lipophilic statin with cumulative contributions of about 93%.

### XGBoost findings

As shown in Fig. 3, the top 5 important features on RM prediction were AST, eGFR, LDL, HTN, and high-dose lipophilic statin. The visualisation of a beeswarm plot indicated that patients with abnormal AST (> 30 U/L), low eGFR level (< 60 mL/min/1.73 m^2^), HTN, and taking high-dose lipophilic statin were higher RM risks, whereas patients with abnormal LDL (> 125 mg/dL) were lower RM risk. The SHAP waterfall plot suggested that cumulative contributions on RM by those five features were about 95%.

### Models performance

**Fig. 4:**
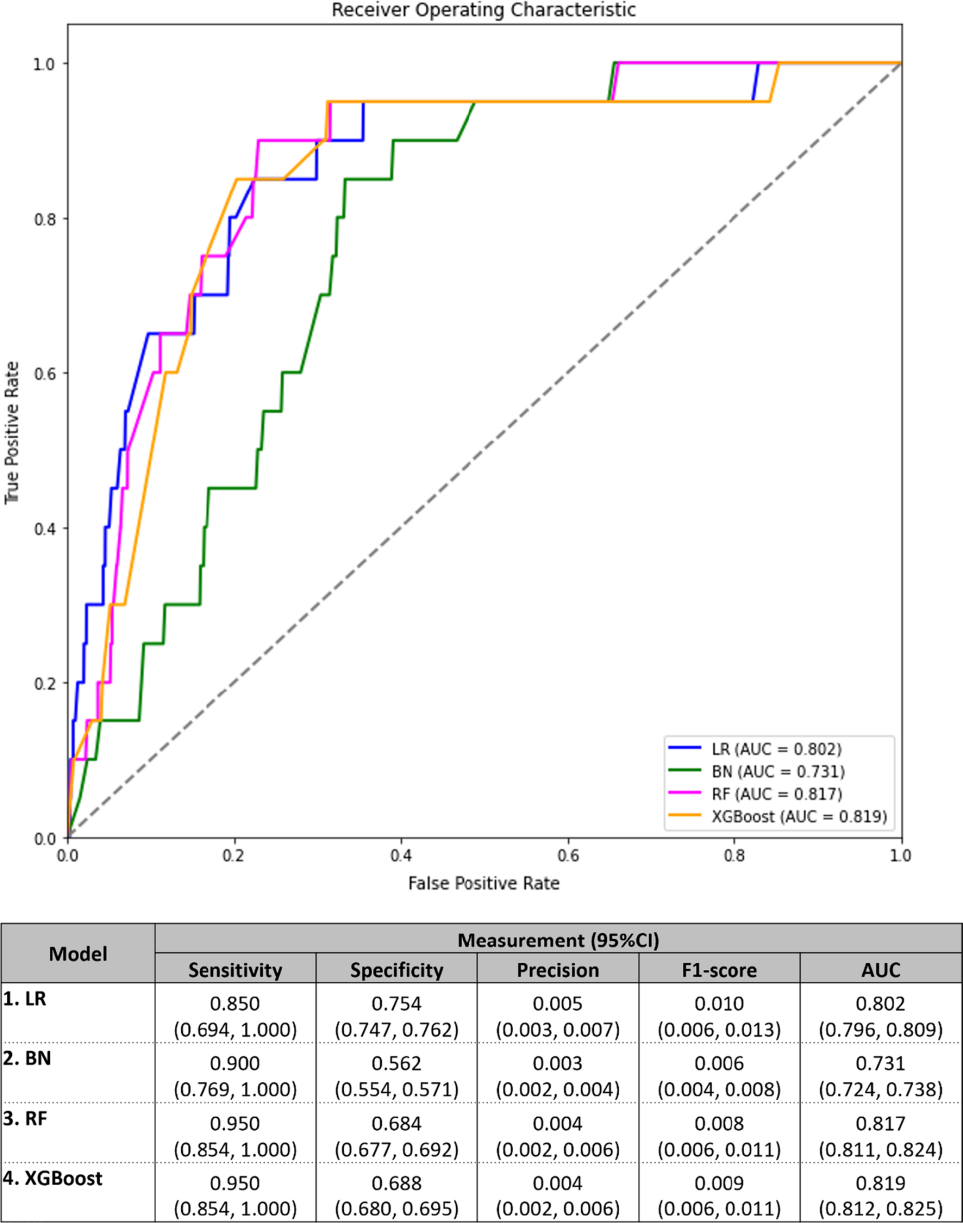
Performance of the proposed models: LR, BN, RF and XGBoost. LR: Logistic regression, BN: Bayesian network, RF: Random forests, XGBoost: Extreme gradient boosting

All performances of LR, BN, RF, and XGBoost were shown in Fig. 4. Sensitivity or recall was the most important metric for this study because the model should correctly detect the occurrence of outcome compared with the actual positive outcome. In addition, the model would be designed as a clinical decision support system which helped to screen the patient. The high number of false negative predictions will affect on loss and delay of effective treatments to patients. As the results, all models had high level of sensitivity (LR: 0.850 (95% CI 0.694, 1.00), BN: 0.900 (95% CI 0.769, 1.000), RF:0.950 (95% CI 0.854, 1.00), XGBoost: 0.950 (95% CI 0.854, 1.00)), but all of them were not significantly different. However, the LR had significantly higher specificity when compared to other models (LR: 0.754 (95% CI 0.747, 0.762), BN: 0.562 (95% CI 0.554, 0.571), RF: 0.684 (95% CI 0.677, 0.692), XGBoost: 0.689 (95% CI 0.680, 0.695)). The area under the ROC was significantly higher in LR [0.802 (95% CI 0.796, 0.809)] than BN [0.731 (95% CI 0.724, 0.738)], but it was significantly slightly lower than the RF [0.817 (95% CI 0.811, 0.824)] and XGBoost [0.819 (95% CI 0.812, 0.825)]. Nonetheless, the precision and F1 score of all models were quite low due to the low prevalence of RM outcome.

Finally, the NRIs (95% CI) comparing BN, RF, and XGBoost with the LR models were − 0.212 (− 0.311, − 0.114), − 0.496 (− 0.594, − 0.397), and − 0.496 (− 0.594, − 0.396), respectively, which were significantly lower than 0 for all NRIs. From that could be interpreted that those three models were significantly inferior in classification than the LR model.

## Discussion

We conducted the study to assess associations of statin use and statin-other drugs interactions with RM and related muscular adverse effects by linkage of electronic databases. The LR model suggested that high-dose lipophilic statin tended to approximate 22.7% higher risk of RM than low-dose hydrophilic statin, but this was not significant. In addition, the high-dose lipophilic statin had interaction effects with clarithromycin about 5.6-folds, and antifungal drugs about 5.8-folds on developing RM, but only clarithromycin was significant. Comparing between explainable-models, the LR was the best relative to BN model, but it was slightly inferior in discriminative performance than the RF and XGBoost with the area under ROCs of 0.802, 0.731, 0.817, and 0.819, respectively. However, the LR model yielded highest F1 score, followed by the XGBoost, RF, and BN, but those were not significantly different. Furthermore, the NRIs indicated that the BN, RF, and XGboost models were significantly inferior in model classifications than the LR model.

The estimated RM prevalence was as high as about 2 per 1000 patients, which was higher than those reported by previous studies [7, 8]. That indicated that data linkages among clinical diagnosis, laboratory findings, and the SRS databases could improve identifying and reporting RM. The process of outcome verification was performed by research teams by reviewing electronic health databases, in order to reduce ascertainment bias of RM outcome [31–33]. All 122 features were listed based on clinical knowledge and review of literatures [3, 34], especially with medications. For developing models, the study had chosen features having high information using several processes of features selection. The bootstrapping technique which was initially applied for screening the relevant features from the high dimensional variables and the collinearity test corresponded to literature [35–37]. Then some drugs were regrouped as new features based on clinical therapeutic uses, i.e., statin groups, antifungals, and NOAC, in order to avoid the problem of zero positive events for each feature. The regrouping decreased granularity of data resulting in in a limitation of drilling down on the details of each drug that belonged to antifungals and NOAC. Finally, both statistical method (univariate analysis) and expert’s judgment were used to consider which features should be included for model development.

The proposed models including LR, BN, RF and XGBoost suggested the association between drugs and the RM with acceptable to high performance levels. Although the sensitivity of the LR model was about 5% and 10% lower than BN and RF/XGBoost models, those were not statistically different. The LR model yielded highest specificity of 0.754 whereas the BN had lowest followed by RF and XGBoost, which were about 19.2%, 7.0%, and 6.6% lower than the LR, respectively. Likewise, the F1 score was highest in the LR model, followed by XGBoost, RF, and BN. However, the area under the ROC of the LR was significantly higher than the BN models [0.805 (0.796, 0.809) vs. 0.731(0.724, 0.738)], respectively, but it was significantly lower than the two other unexplainable models of RF and XGBoost [0.817 (0.811, 0.824) and 0.819 (0.812, 0.825)], respectively. The LR and BN models were known as the white-box models, which could estimate the effect size of features on RM directly. Although the RF and XGBoost were known as the black-box models (i.e., unable to estimate effect sizes), feature importance and contribution on RM outcome could be explained by the SHAP values. However, the NRIs indicated that BN, RF, and XGBoost were lower performance in misclassifying non-RM than the LR model. In addition, interpretation and explainability of the model played an important role in healthcare domain, because the impact of features on RM should be relevant and correspond to biological/clinical mechanism and knowledge leading awareness in drug prescription. Therefore, the LR should be more suitable to the aims of this study and also be useful in clinical interpretation and more applicable than the BN, RF and XGBoost.

For the results of LR, a higher odds of outcome with taking a high-dose lipophilic statin corresponded to the characteristics of statins [9, 38, 39]. In accordance with the higher odds of outcome with concurrent use of high-dose lipophilic statin and another drug were related to the metabolic and elimination pathway of statins. Both substrates (ticagrelor and colchicine) and inhibitors (antifungals, clarithromycin, and cyclosporine) of CYP3A4 were competitors in the metabolic pathway of simvastatin, lovastatin, and atorvastatin. Carvedilol is an inhibitor of P-glycoprotein, a cellular transport pathway of all statins. Moreover, concurrent use of those drugs (e.g., cyclosporin, digoxin, ticagrelor, and antifungal) with hydrophilic statin also remarkably increased the conditional probabilities of the outcome in accordance with results of BN. Therefore, these medications induced statin retention leading to higher odds of the outcome corresponding to previous studies [31–33, 40–43]. In addition, a patient who had been concurrently taking high-dose lipophilic statins, clarithromycin, and antifungals should be closely monitored about the outcome due to the high odds of outcome. Additionally, patients who had hypertension, elevated AST level, or decreased eGFR level were higher risk of RM which corresponded to literature [3].

For clinical implementation, the proposed LR model should be applied for confirmation of the outcome occurrence, because it had a higher performance as well as easy for use and explanation with an explicit coefficient of an individual feature. Nevertheless, LR was not suitable to use as a clinical decision supporting system due to several limitations of multiple logit regression models [44]. First, the logit model requires all known variables for prediction. Unfortunately, missing data always happens in real clinical practice, and the laboratory features are also usually reported after the outcome has occurred. Also, interaction effects analysis is done by manually fitting the interaction terms into the model, so the LR model is inconveniently used for the analysis of interaction effects that contain more than 2 variables. On the contrary, the BN model could be applied for screening the outcome before happening because it could solve those limitations for several reasons. First, the model predicts the outcome based on prior knowledge learned from the train dataset and the given evidence, so the model can handle missing data. Second, the interaction effects analysis could be done by feeding the conditions as a new dataset or directly inputting evidence into the interactive model. Hence, the model could predict all input interaction terms at once resulting in less time consumption and more comfort compared to the logit model. As a result, the BN could find the worst scenario of patients who would have the highest probability of an outcome after analysing all possible interaction effects. Moreover, the BN model allows immediate prediction of the conditional probability of the outcome for individual patients.

Nonetheless, there were several limitations of the study as follows: First, the data was retrieved from one hospital in Thailand resulting in a low prevalence of the outcome that could affect on the reliability and accuracy of the model. To avoid the problem of zero events, the study had regrouped some drug features, which led to another limitation of data granularity. For example, the model could not drill down on the details of whether the active ingredient, i.e., simvastatin, atorvastatin, pravastatin, fluvastatin, rosuvastatin, or pitavastatin can actually cause the outcome. Second, there was an incomplete diagnosis recorded in the clinical databases resulting in the problems of outcome identification. Some events were missing because the ICD-10-TM code for rhabdomyolysis has only emerged since 2016. Manual chart reviews were executed to solve this gap. However, if there is a higher rate of completeness and correctness of recording ICD-10-TM in the future, then manual chart reviews might be unnecessary. Third, the process of data preparation was complex and complicated due to silos of data. All data obtained from each electronic database had to be merged together for preparing the longitudinal data of individual patients. The study included almost 140,000 statin users with more than 1.5 million prescriptions of statins. Merging all data, building individual patient’s timeline, and mapping features to the timelines were time-consuming and high computation was needed. Also, the BN model should update the prior knowledge regularly to accordingly accommodate changes in clinical practice. Therefore, a technique of stored procedure should be used for more practical data preparation. The stored procedure is a prepared query coding that is created for automatic data preparation and saved in the databases without increasing the size of data. Fourth, although we had attempted to identify interaction of statin-other drugs, and also interaction among other drugs themselves, only few significant interactions of statin-other drugs could be identified, whereas the rest of statin-other drugs interactions (i.e., ticagrelor, cyclyosporine, carvedilol, and colchicine) only showed trend of associations due to small numbers of RM outcomes. Our study also considered other drug-drug interactions, but this caused overfitting of the model, so they were omitted. Neither pharmacokinetic nor genetic feature was routinely assessed, thus they were not considered. As a result, this study could only repeat previous knowledge without adding new knowledge from the literature.

## Conclusion

Statin use with high-dose lipophilic might have drug interactions with others including clarithromycin and antifungals. Use of electronic data linkages should be helpful in identify RM cases from statins and those medications. A prospective evaluation of the model should be further assessed with well planned data monitoring for drug prescriptions and relevant laboratory tests particularly for CK level leading to validating the LR model. Application of the LR model should then be developed later to plug them into a routine drug prescription system.

## Supplementary Information

**Additional file 1**. Table. A1.

**Additional file 2**. Fig. A1.

## Data Availability

The authors confirm that the data supporting the findings of this study are available within the article and its supplementary materials.
