# Supplementary material for: Detection of statin-induced rhabdomyolysis and muscular related adverse events through data mining technique
Source: BMC Med Inform Decis Mak. 2022 Sep 5;22:233. doi: 10.1186/s12911-022-01978-4 (PMC9446837; doi:10.1186/s12911-022-01978-4)
Supplement: Supplementary file 1 — Additional file 1. Table. A1. [file 12911_2022_1978_MOESM1_ESM.docx]

**Appendix Tables**

Table A.1 List of all extracted features

Table A.2 List of drugs extracted from the medication database

Table A.3 List of extracted laboratory tests

Table A.4 Clinical diagnosis and procedures extracted to the study

Table A.5 Conditions for mapping features

Table A.6 Entry features and their possible values

Table A.7 Characteristic of data before imputations

Table A.8 Distribution of imputed features

Table A.9 Data distribution over the discretised features

Table A.10 List of included features and their possible values

Table A.11 The conditional probability of an outcome given features

Table A.12 Conditional probabilities of the outcome (%) predicted from concurrent using low-dose hydrophilic statins with other 2 drugs

Table A.13 Conditional probabilities of the outcome (%) predicted from concurrent using high-dose hydrophilic statins with other 2 drugs

Table A.14 Conditional probabilities of the outcome (%) predicted from concurrent using low-dose lipophilic statins with other 2 drugs

Table A.15 Conditional probabilities of the outcome (%) predicted from concurrent using high-dose lipophilic statins with other 2 drugs

Table A.16 The highest predicted probability of an outcome based on a group of statin users

**Table A.1 List of all extracted features**

| **Database** | **Features extraction** |
| --- | --- |
| **1. Registration** | **Demographic data**  1. Date of birth  2. Sex |
| **2. Medication**  (listed in Table A.2) | **Medications**  1. Prescriptions of statins  2. Prescriptions of drugs increasing the level of statins  3. Prescriptions of other drugs induced RM |
| **3. Laboratory chemistry**  (listed in Table A.3) | **Laboratory findings**  1. Creatinine kinase (CK)  2. Liver function test (AST)  3. Renal function test (S_Cr_)  4. Renal functional stages (eGFR)  5. Electrolytes (potassium, phosphate, calcium, sodium)  6. Lipid profile (TC, HDL, TG, LDL) |
| **4. Diagnosis**  (listed in Table A.4) | **Underlying diseases**  1. Dyslipidemia  2. Chronic heart failure  3. Hypertension  4. Fibrosis and cirrhosis of liver |
|  | **Comorbidities**  1. Hyperaldosteronism  2. Thyroid disorders  3. Diabetic ketoacidosis  4. Glucocorticoid disorders  5. Vitamin D deficiency  6. Acromegaly  7. Hypopituitarism  8. Stroke  9. Multiple Sclerosis  10. Guillain-Barre Syndrome (GBS)  11. Neuromuscular complications of diabetes mellitus  12. Myasthenia gravis  13. Cervical spondylosis  14. Degenerative disc disease  15. Human immunodeficiency viruses (HIV)  16. Epstein-Barr Virus (EVB) infectious |
|  | **Diagnosis used for positive outcome identification**  *Inclusion criteria of positive outcome identification*  1. Myalgia  2. Drug-induced myopathy  3. Myopathy due to other toxic agents  4. Rhabdomyolysis  *Exclusion criteria of positive outcome identification*  1. Diagnosis MI  2. Procedure for treatment MI  3. Dermatopolymyositis |
| **5. Spontaneous Reporting System (SRS)** | Reports of rhabdomyolysis |

**Table A.2 List of drugs extracted from the medication database**

| **Group** | **Drug Name** | **TMTID**  **(SUBS)** | **Fully Specified Name (FSN)** |
| --- | --- | --- | --- |
| **1. Statins** | | | |
| **1.1 Lipophilic** | Atorvastatin | 223312 | atorvastatin |
|  |  | 223320 | atorvastatin calcium |
|  | Fluvastatin | 224218 | fluvastatin |
|  | Pitavastatin | 225261 | pitavastatin calcium |
|  | Simvastatin | 225411 | simvastatin |
| **1.2 Hydrophilic** | Pravastatin | 225288 | pravastatin sodium |
|  | Rosuvastatin | 225392 | rosuvastatin |
| **2. Drugs increasing level of statins.** | | | |
| **2.1 CYP2C9** | **Sensitive substrates** | | |
|  | Celecoxib | 223618 | celecoxib |
|  | **Moderate sensitive substrates** | | |
|  | Glimepiride | 237030 | glimepiride |
|  | Phenytoin^(d)^ | 301691 | phenytoin |
|  |  | 769585 | phenytoin sodium |
|  | Tolbutamide | N/A | tolbutamide |
|  | Warfarin | 225929 | warfarin sodium |
|  | **Moderate inhibitors** | | |
|  | Amiodarone^(c)^ | 765203 | amiodarone hydrochloride |
|  | Fluconazole^(f)^ | 263820 | fluconazole |
|  | Miconazole | N/A | miconazole |
|  | **Weak inhibitors** | | |
|  | Diosmin | 737480 | diosmin |
| **2.2 CYP3A4** | **Sensitive substrates** | | |
|  | Darunavir | 648647 | darunavir |
|  | Everolimus | 224084 | everolimus |
|  | Midazolam | 533901 | midazolam^(g)^ |
|  | Saquinavir | 330683 | saquinavir |
|  | Sirolimus | 225424 | sirolimus |
|  |  | 225430 | sirolimus nanosystems dispersion |
|  | Tacrolimus^(a)^ | 225566 | tacrolimus |
|  | Vardenafil | 358753 | vardenafil |
|  | Budesonide | 269694 | budesonide |
|  | Dasatinib | 223832 | dasatinib |
|  | Dronedarone^(b), (c)^ | 383753 | dronedarone |
|  | Eletriptan | 424408 | eletriptan |
|  | Felodipine | 224124 | felodipine |
|  | Indinavir | 658370 | indinavir |
|  | Lurasidone | 1008800 | lurasidone hydrochloride |
|  | Maraviroc | 644011 | maraviroc |
|  | Quetiapine | 421995 | quetiapine |
|  | Sildenafil | 649324 | sildenafil |
|  | Ticagrelor^(a)^ | 225964 | ticagrelor^(a)^ |
|  | Tolvaptan | 698477 | tolvaptan |
|  | **Moderate sensitive substrates** | | |
|  | Alprazolam | 233068 | alprazolam^(g)^ |
|  | Aprepritant^(b)^ | 321058 | aprepitant |
|  | Colchicine | 279254 | colchicine |
|  | Pimozide | 398910 | pimozide |
|  | Rilpivirine | 674473 | rilpivirine |
|  | Rivaroxaban | 651832 | rivaroxaban |
|  | Tadalafil | 281822 | tadalafil |
|  | **Strong inhibitors** | | |
|  | Clarithromycin^(c)^ | 263094 | clarithromycin |
|  | Cobicistat | 966002 | cobicistat |
|  | Diltiazem | 223897 | diltiazem hydrochloride |
|  | Itraconazole^(c)^ | 341304 | itraconazole |
|  | Ketoconazole | 262599 | ketoconazole |
|  | Posaconazole | 660289 | posaconazole |
|  | Ritonavir^(c)^ | 236558 | ritonavir |
|  | Voriconazole^(e)^ | 651536 | voriconazole |
|  | **Moderate inhibitors** | | |
|  | Cimetidine | 223706 | cimetidine |
|  | Ciprofloxacin | 266337 | ciprofloxacin |
|  | Clotrimazole | 266183 | clotrimazole |
|  | Crizotinib | 653847 | crizotinib |
|  | Cyclosporine | 772624 | cyclosporine |
|  | Erythromycin | 322408 | erythromycin |
|  |  | 855804 | erythromycin stearate |
|  | Fluvoxamine^(e)^ | 738171 | fluvoxamine |
|  |  | 645818 | fluvoxamine maleate |
|  | Imatinib | 224434 | imatinib |
|  | Verapamil^(c)^ | 990797 | verapamil hydrochloride |
|  | **Weak inhibitors** | | |
|  | Cilostazol | 223694 | cilostazol |
|  | Fosaprepitant | 695992 | fosaprepitant |
|  |  | 964140 | fosaprepitant dimeglumine |
|  | Ranitidine | 225344 | ranitidine |
| **2.3 Glucuronidation** | **Substrates** | | |
|  | Entacapone | 293530 | entacapone |
|  | Gemfibrozil | 224287 | gemfibrozil^(g)^ |
|  | Indomethacin | 999834 | indometacin |
|  | lamotrigine | 356662 | lamotrigine |
|  | Telmisartan | 225597 | telmisartan |
|  | Zidovudine | 264049 | zidovudine |
|  | **Inhibitors** | | |
|  | Amitriptyline | 238883 | amitriptyline hydrochloride^(g)^ |
|  | Chlorpromazine | 236799 | chlorpromazine hydrochloride^(g)^ |
|  | Clomipramine | 642837 | clomipramine hydrochloride |
|  | Clonazepam | 245779 | clonazepam^(g)^ |
|  | Diazepam | 255953 | diazepam^(g)^ |
|  | Ethinylestradiol | 679508 | ethinylestradiol |
|  | Flunitrazepam | 534528 | flunitrazepam^(g)^ |
|  | Ibuprofen | 224391 | ibuprofen |
|  | Imipramine | 650705 | imipramine hydrochloride |
|  | Ketoprofen | 224605 | ketoprofen |
|  | Lorazepam | 246663 | lorazepam^(g)^ |
|  | Naproxen | 224959 | naproxen |
|  | Nitrazepam | 534456 | nitrazepam^(g)^ |
|  | Probenecid | 259937 | probenecid |
|  | Promethazine | 531736 | promethazine hydrochloride^(g)^ |
|  | Propranolol | 235753 | propranolol hydrochloride |
|  | Sertraline | 652196 | sertraline |
| **2.4 P-glycoprotein** | **Substrates** | | |
|  | Dabigatran | 648407 | dabigatran etexilate |
|  | Digoxin | 273028 | digoxin |
|  | Fexofenadine | 974347 | fexofenadine hydrochloride |
|  | **Inhibitors** | | |
|  | Carvedilol | 272226 | carvedilol |
|  | Lapatinib | 224633 | lapatinib |
|  | Ranolazine | 996547 | ranolazine |
| **3. Other drugs induced RM** | | | |
| **3.1 Other lipid-lowering agents** | Bezafibrate | 223417 | bezafibrate |
|  | Ezetimibe | 224107 | ezetimibe |
| **3.2 Psychiatric agents** | Doxepin | 443197 | doxepin |
|  | Fluoxetine | 230343 | fluoxetine |
|  | Fluphenazine | 278225 | fluphenazine |
|  |  | 764434 | fluphenazine decanoate |
|  | Haloperidol | 331121 | haloperidol |
|  |  | 763907 | haloperidol decanoate |
|  | Lithium | 737904 | lithium carbonate |
|  | Perphenazine | 247634 | perphenazine |
|  | Trifluoperazine | 325319 | trifluoperazine |
|  | Venlafaxine | 320320 | venlafaxine |
|  | **Benzodiazepines** |  |  |
|  | Bromazepam | 533363 | bromazepam |
|  | Chlordiazepoxide | 260707 | chlordiazepoxide |
|  | Clobazam | 331262 | clobazam |
|  | Dipotassium clorazepate | 532593 | dipotassium clorazepate |
|  | Flurazepam | N/A | flurazepam |
|  | Temazepam | N/A | temazepam |
|  | **Barbiturates** |  |  |
|  | Pentobarbital | 530958 | pentobarbital sodium |
|  | Phenobarbital | 373192 | phenobarbital |
|  |  | 533226 | phenobarbital sodium |
| **3.3 Other** | Diphenhydramine | 244969 | diphenhydramine |
|  |  | 748427 | diphenhydramine hydrochloride |
|  | Amphotericin B | 655095 | amphotericin b |
|  | Arsenic | 223298 | arsenic trioxide |
|  | Azathioprine | 223349 | azathioprine |
|  | Halothane | N/A | halothane |
|  | Naltrexone | N/A | naltrexone |
|  | Quinidine | N/A | quinidine |
|  | Penicillamine | 225203 | penicillamine |
|  | Pentamidine | 660918 | pentamidine isethionate |
|  | Propofol | 523629 | propofol |
|  | Salicylates | 225407 | salicylic acid |
|  | Succinylcholine | 723402 | suxamethonium chloride |
|  | Theophylline | 231771 | theophylline |
|  | Terbutaline | 859544 | terbutaline sulfate |
|  | **Thiazides** | | |
|  | Bendroflumethiazide | N/A | bendroflumethiazide |
|  | Hydrochlorothiazide | 224362 | hydrochlorothiazide |
|  | Vasopressin | 572273 | vasopressin |

^(a)^ The drug also has a weak inhibitory effect on CYP3A4.

^(b)^ The drug also has a moderate inhibitory effect on CYP3A4.

^(c)^ The drug also has an inhibitory effect on P-glycoprotein.

^(d)^ The drug also has an inhibitory effect on glucuronidation.

^(e)^ The drug also has a weak inhibitory effect on CYP2C9.

^(f)^ Fluconazole has a moderate inhibitory effect on CYP2C9 and CYP3A4. However, a study found that fluconazole significantly interacted with fluvastatin (27), categorised into CYP2C9 inhibitors for this study.

^(g)^ The drug also can induce RM directly according to literature (3).

**Table A.3 List of extracted laboratory tests**

| **Group** | **Lab Test** | **Codes ^(a)^** | **TMLT** | **TMLT Name** |
| --- | --- | --- | --- | --- |
| **CK** | Creatine kinase | 200054 | 320123 | Creatine kinase [U/L] in Serum or Plasma |
| **Liver function test** | AST | 200023 | 320150 | Aspartate aminotransferase [U/L] in Serum or Plasma |
| **Renal function test** | Serum creatinine (S_Cr_) | 200058, 201080 | 320055 | Creatinine [mg/dL] in Serum or Plasma |
|  | Estimated glomerular filtration rate (eGFR) | 200718 | N/A | N/A |
| **Electrolytes** | Potassium | 200122 | 320026 | Potassium [mmol/L] in Serum or Plasma |
|  | Phosphate | 200090 | 320022 | Phosphate [mg/dL] in Serum or Plasma |
|  | Calcium | 200033 | 320001 | Calcium [mg/dL] in Serum or Plasma |
|  | Sodium | 200140 | 320032 | Sodium [mmol/L] in Serum or Plasma |
| **Lipid profile** | Total cholesterol (TC) | 200047 | 320070 | Cholesterol [mg/dL] in Serum or Plasma |
|  | High-density lipoproteins (HDL) | 200085 | 320071 | Cholesterol in HDL [mg/dL] in Serum or Plasma |
|  | Triglyceride (TG) | 200157 | 320072 | Triglyceride [mg/dL] in Serum or Plasma |
|  | Low-density lipoprotein (LDL) | 200106 | 320073 | Cholesterol in LDL [mg/dL] in Serum or Plasma by Direct assay |

^(a)^ Hospital codes

**Table A.4 Clinical diagnosis and procedures extracted to the study**

| **Diseases** | **ICD-10-TM / ICD-9-CM / Hospital codes** |
| --- | --- |
| **1. Underlying diseases** | |
| 1.1 Dyslipidemia | E78 |
| 1.2 Chronic heart failure | I50 |
| 1.3 Hypertension | I10, I15 |
| 1.4 Fibrosis and cirrhosis of liver | K74 |
| 1.5 Coronary artery disease | I25 |
| **2. Comorbidities** | |
| 2.1 Hyperaldosteronism | E26 |
| 2.2 Thyroid disorders | E00 - E05 |
| 2.3 Diabetic ketoacidosis | E10.1, E11.1, E12.1, E13.1, E14.1 |
| 2.4 Glucocorticoid disorders | E24, E25, E27 |
| 2.5 Vitamin D deficiency | E55 |
| 2.6 Acromegaly | E22 |
| 2.7 Hypopituitarism | E23 |
| 2.8 Stroke | I60-I64 |
| 2.9 Multiple Sclerosis | G35 |
| 2.10 Guillain-Barre Syndrome (GBS) | G61 |
| 2.11 Neuromuscular complications of diabetes mellitus | E10.4, E11.4, E12.4, E13.4, E14.4, E16.4 |
| 2.12 Myasthenia gravis | G70 |
| 2.13 Cervical spondylosis | M47.02, M47.12, M47.22, M47.82, M47.92 |
| 2.14 Degenerative disc disease | M51 |
| 2.15 HIV | B20 - B24 |
| 2.16 Epstein-Barr Virus (EVB) infectious | D82.3 |
| **3. Outcome identification** | |
| **3.1 Inclusion criteria for positive outcome** | |
| 3.1.1 Myalgia | M79.1 |
| 3.1.2 Drug-induced myopathy | G72.0 |
| 3.1.3 Myopathy due to other toxic agents | G72.2 |
| 3.1.4 Rhabdomyolysis | M62.7 |
| 3.1.5 Reports of rhabdomyolysis in SRS | 121000100740200 |
| **3.2 Exclusion criteria for positive outcome** | |
| 3.2.1 Diagnosis of MI | I21-I24 |
| 3.2.2 Dermatomyositis and polymyositis | M33 |
| 3.2.3 Procedure for treatment MI | 36.0, 36.1, 00.66 |

**Table A.5 Conditions for mapping features**

| **Type of outcome and criteria** | **Type of features** |
| --- | --- |
| **Positive outcome** |  |
| 1. A feature was included if it had been recorded within 1 month before the occurrence of the outcome. | - Comorbidities - Medications |
| 2. A feature was included if it had been recorded within 1 month before the occurrence of the outcome until 7 days after that. | - Laboratory findings |
| **Negative outcome** |  |
| A feature was included if had been recorded within the period of the event. | All types of features |

**Table A.6 Entry features and their possible values**

| **Features** | | | | **Possible values** | | | | | |
| --- | --- | --- | --- | --- | --- | --- | --- | --- | --- |
| **Demographic data: 2** | | | |  | | | | | |
| 1. Sex | | | | 0: male, 1: female | | | | | |
| 2. Age | | | | 0: < 65 years, 1: ≥ 65 years | | | | | |
| **Interested medications: 3** | | | |  | | | | | |
| 1. Type of treatment | | | | 0: statin alone, 1: combinations | | | | | |
| 2. Lipophilic or Hydrophilic | | | | 0: hydrophilic, 1: lipophilic | | | | | |
| 3. Dose of statins | | | | 0: standard dose, 1: high dose | | | | | |
| 4. Drugs increasing statin level: 77 | | | | 0: absent, 1: coadministration (for each drug): | | | | | |
| Amiodarone | Aprepritant | | | | Budesonide | Carvedilol | | | Celecoxib |
| Cilostazol | Cimetidine | | | | Ciprofloxacin | Clarithromycin | | | Clomipramine |
| Cobicistat | Colchicine | | | | Crizotinib | Cyclosporine | | | Dabigatran |
| Darunavir | Dasatinib | | | | Digoxin | Diltiazem | | | Diosmin |
| Dronedarone | Eletriptan | | | | Entacapone | Erythromycin | | | Ethinylestradiol |
| Everolimus | Felodipine | | | | Fexofenadine | Fluconazole | | | Fluvoxamine |
| Fosaprepitant | Glimepiride | | | | Ibuprofen | Imatinib | | | Imipramine |
| Indomethacin | Itraconazole | | | | Ketoconazole | lamotrigine | | | Lapatinib |
| Lurasidone | Naproxen | | | | Phenytoin | Pimozide | | | Posaconazole |
| Probenecid | Propranolol | | | | Quetiapine | Ranitidine | | | Ranolazine |
| Rilpivirine | Ritonavir | | | | Rivaroxaban | Saquinavir | | | Sertraline |
| Sildenafil | Sirolimus | | | | Tacrolimus | Tadalafil | | | Telmisartan |
| Ticagrelor | Tolvaptan | | | | Vardenafil | Verapamil | | | Voriconazole |
| Warfarin | Zidovudine | | | | Alprazolam | Amitriptyline | | | Chlorpromazine |
| Clonazepam | Diazepam | | | | Flunitrazepam | Gemfibrozil | | | Lorazepam |
| Midazolam | Nitrazepam | | | |  |  | | |  |
| 4. Other drugs induced RM:26 | | | | 0: not coadministration, 1: coadministration (for each drug) | | | | | |
| Amphotericin B | Azathioprine | | | | Bezafibrate | Bromazepam | | | Chlordiazepoxide |
| Chlordiazepoxide | Clobazam | | | | Clorazepate | Diphenhydramine | | | Doxepin |
| Ezetimibe | Fluoxetine | | | | Fluphenazine | Haloperidol | | | Hydrochlorothiazide |
| Lithium | Penicillamine | | | | Pentamidine | Perphenazine | | | Phenobarbital |
| Propofol | Salicylates | | | | Succinylcholine | Terbutaline | | | Theophylline |
| Trifluoperazine |  | | | |  |  | | |  |
| **Laboratory findings:8** | | | |  | | | | | |
| 1. AST | | | | 0: normal (≤30 U/L), 1: abnormal (>30 U/L) | | | | | |
| 2. eGFR | | | | 0: eGFR ≥60 mL/min/1.73m^2^, 1: eGFR <60 mL/min/1.73m^2^ | | | | | |
| 3. K | | | | 0: normal (≥3.5 mmol/L), 1: abnormal (<3.5 mmol/L) | | | | | |
| 4. PO_4_ | | | | 0: normal (≥2.5 mg/dL), 1: abnormal (<2.5 mg/dL) | | | | | |
| 5. Ca | | | | 0: normal (≥8.5 mg/dL), 1: abnormal (<8.5 mg/dL) | | | | | |
| 6. Na | | | | 0: normal (135-145 mmol/L)  1: abnormal (<135 or >145 mmol/L) | | | | | |
| 7. LDL | | | | 0: normal (≤125), 1: abnormal (>125) | | | | | |
| 8. TG | | | | 0: normal (≤150 mg/dL), 1: abnormal (>150 mg/dL) | | | | | |
| **Underlying diseases 5** | | | | 0: not-having, 1: having the disease | | | | | |
| Dyslipidemia | | Chronic heart failure | | | | | Hypertension | | |
| Fibrosis and cirrhosis of liver | | Coronary artery diseases | | | | |  | | |
| **Comorbidities^(a)^** | | | | 0: not-having, 1: having disease 16 | | | | | |
| Hyperaldosteronism | | | Thyroid disorders | | | | | Diabetic ketoacidosis | |
| Glucocorticoid disorders | | | Vitamin D deficiency | | | | | Acromegaly | |
| Hypopituitarism | | | Stroke | | | | | Multiple Sclerosis | |
| Guillain-Barre Syndrome (GBS) | | | Myasthenia gravis | | | | | Cervical spondylosis | |
| Degenerative disc disease | | | Human immunodeficiency viruses (HIV) | | | | | | |
| Epstein-Barr Virus (EVB) infectious | | | | | | | |  | |
| Neuromuscular complications of diabetes mellitus | | | | | | | |  | |

^(a)^ If at least one of these comorbidities was diagnosed, the variable was coded as 1.

**Table A.7 Characteristics of data before imputations**

| **Features** | **Original** | | **Train** | | **Test** | |
| --- | --- | --- | --- | --- | --- | --- |
|  | **N (70,470)** | **%** | **N (56,377)** | **%** | **N (14,093)** | **%** |
| **OUTCOME** | | | | | | |
| Negative | 70,366 | 99.85 | 56,293 | 99.85 | 14,073 | 99.86 |
| Positive | 104 | 0.15 | 84 | 0.15 | 20 | 0.14 |
| **Demographic** | | | | | | |
| Age, mean(SD) | 67.18 (12.05) | | 67.20 (12.00) | | 67.10 (12.27) | |
| Sex |  |  |  |  |  |  |
| Male | 25,796 | 36.61 | 20,647 | 36.62 | 5,149 | 36.54 |
| Female | 44,674 | 63.39 | 35,730 | 63.38 | 8,944 | 63.46 |
| **Comorbidity** | | | | | | |
| Absent | 58,726 | 83.33 | 47,025 | 83.41 | 11,701 | 83.03 |
| Present | 11,744 | 16.67 | 9,352 | 16.59 | 2,392 | 16.97 |
| **Underlying diseases** | | | | | | |
| Hypertension |  |  |  |  |  |  |
| Absent | 27,889 | 39.58 | 22,352 | 39.65 | 5,537 | 39.29 |
| Present | 42,581 | 60.42 | 34,025 | 60.35 | 8,556 | 60.71 |
| Coronary artery diseases |  |  |  |  |  |  |
| Absent | 56,976 | 80.85 | 45,569 | 80.83 | 11,407 | 80.94 |
| Present | 13,494 | 19.15 | 10,808 | 19.17 | 2,686 | 19.06 |
| Chronic heart failure |  |  |  |  |  |  |
| Absent | 67,034 | 95.12 | 53,632 | 95.13 | 13,402 | 95.10 |
| Present | 3,436 | 4.88 | 2,745 | 4.87 | 691 | 4.90 |
| Fibrosis and cirrhosis of liver |  |  |  |  |  |  |
| Absent | 69,631 | 98.81 | 55,693 | 98.79 | 13,938 | 98.90 |
| Present | 839 | 1.19 | 684 | 1.21 | 155 | 1.10 |
| **Statin group** |  |  |  |  |  |  |
| Hydrophilic | 8,791 | 12.47 | 7,037 | 12.48 | 1,754 | 12.45 |
| Lipophilic | 61,679 | 87.53 | 49,340 | 87.52 | 12,339 | 87.55 |
| **Statin dose** | | | | | | |
| Number of DDD, median(range) | 0.67  (0.04, 2442.00) | | 0.67  (0.04, 2442.00) | | 0.67  (0.07, 182.00) | |
| Statin alone or combinations |  |  |  |  |  |  |
| Use statin alone | 25,141 | 35.68 | 20,134 | 35.71 | 5,007 | 35.53 |
| Use combinations | 45,329 | 64.32 | 36,243 | 64.29 | 9,086 | 64.47 |
| **Drugs increasing level of statins** | | | | | | |
| **CYP2C9** | | | | | | |
| **CYP2C9 substrates** | | | | | | |
| Celecoxib |  |  |  |  |  |  |
| Absent | 66,027 | 93.70 | 52,820 | 93.69 | 13,207 | 93.71 |
| Present | 4,443 | 6.30 | 3,557 | 6.31 | 886 | 6.29 |
| Glimepiride |  |  |  |  |  |  |
| Absent | 69,960 | 99.28 | 55,971 | 99.28 | 13,989 | 99.26 |
| Present | 510 | 0.72 | 406 | 0.72 | 104 | 0.74 |
| Phenytoin |  |  |  |  |  |  |
| Absent | 69,846 | 99.11 | 55,876 | 99.11 | 13,970 | 99.13 |
| Present | 624 | 0.89 | 501 | 0.89 | 123 | 0.87 |
| Warfarin |  |  |  |  |  |  |
| Absent | 66,838 | 94.85 | 53,472 | 94.85 | 13,366 | 94.84 |
| Present | 3,632 | 5.15 | 2,905 | 5.15 | 727 | 5.16 |
| **CYP2C9 inhibitors** | | | | |  |  |
| Amiodarone |  |  |  |  |  |  |
| Absent | 67,991 | 96.48 | 54,348 | 96.40 | 13,643 | 96.81 |
| Present | 2,479 | 3.52 | 2,029 | 3.60 | 450 | 3.19 |
| Diosmin |  |  |  |  |  |  |
| Absent | 68,539 | 97.26 | 54,807 | 97.22 | 13,732 | 97.44 |
| Present | 1,931 | 2.74 | 1,570 | 2.78 | 361 | 2.56 |
| Fluconazole |  |  |  |  |  |  |
| Absent | 69,976 | 99.30 | 55,980 | 99.30 | 13,996 | 99.31 |
| Present | 494 | 0.70 | 397 | 0.70 | 97 | 0.69 |
| **CYP3A4** | | | | |  |  |
| **CYP3A4 moderate sensitive substrates** | | | | |  |  |
| Alprazolam |  |  |  |  |  |  |
| Absent | 65,366 | 92.76 | 52,293 | 92.76 | 13,073 | 92.76 |
| Present | 5,104 | 7.24 | 4,084 | 7.24 | 1,020 | 7.24 |
| Aprepitant |  |  |  |  |  |  |
| Absent | 70,443 | 99.96 | 56,355 | 99.96 | 14,088 | 99.96 |
| Present | 27 | 0.04 | 22 | 0.04 | 5 | 0.04 |
| Colchicine |  |  |  |  |  |  |
| Absent | 67,476 | 95.75 | 53,977 | 95.74 | 13,499 | 95.79 |
| Present | 2,994 | 4.25 | 2,400 | 4.26 | 594 | 4.21 |
| Pimozide |  |  |  |  |  |  |
| Absent | 70,457 | 99.98 | 56,368 | 99.98 | 14,089 | 99.97 |
| Present | 13 | 0.02 | 9 | 0.02 | 4 | 0.03 |
| Rilpivirine |  |  |  |  |  |  |
| Absent | 70,446 | 99.97 | 56,357 | 99.96 | 14,089 | 99.97 |
| Present | 24 | 0.03 | 20 | 0.04 | 4 | 0.03 |
| Rivaroxaban |  |  |  |  |  |  |
| Absent | 69,890 | 99.18 | 55,914 | 99.18 | 13,976 | 99.17 |
| Present | 580 | 0.82 | 463 | 0.82 | 117 | 0.83 |
| Tadalafil |  |  |  |  |  |  |
| Absent | 70,378 | 99.87 | 56,306 | 99.87 | 14,072 | 99.85 |
| Present | 92 | 0.13 | 71 | 0.13 | 21 | 0.15 |
| **CYP3A4 sensitive substrates** | | | | | | |
| Budesonide |  |  |  |  |  |  |
| Absent | 70,469 | 100 | 56,376 | 100 | 14,093 | 100 |
| Present | 1 | 0 | 1 | 0 | 0 | 0 |
| Darunavir |  |  |  |  |  |  |
| Absent | 70,460 | 99.99 | 56,368 | 99.98 | 14,092 | 99.99 |
| Present | 10 | 0.01 | 9 | 0.02 | 1 | 0.01 |
| Dasatinib |  |  |  |  |  |  |
| Absent | 70,464 | 99.99 | 56,374 | 99.99 | 14,090 | 99.98 |
| Present | 6 | 0.01 | 3 | 0.01 | 3 | 0.02 |
| Dronedarone |  |  |  |  |  |  |
| Absent | 70,441 | 99.96 | 56,353 | 99.96 | 14,088 | 99.96 |
| Present | 29 | 0.04 | 24 | 0.04 | 5 | 0.04 |
| Eletriptan |  |  |  |  |  |  |
| Absent | 70,400 | 99.9 | 56,315 | 99.89 | 14,085 | 99.94 |
| Present | 70 | 0.1 | 62 | 0.11 | 8 | 0.06 |
| Everolimus |  |  |  |  |  |  |
| Absent | 70,376 | 99.87 | 56,306 | 99.87 | 14,070 | 99.84 |
| Present | 94 | 0.13 | 71 | 0.13 | 23 | 0.16 |
| Felodipine |  |  |  |  |  |  |
| Absent | 69,010 | 97.93 | 55,202 | 97.92 | 13,808 | 97.98 |
| Present | 1,460 | 2.07 | 1,175 | 2.08 | 285 | 2.02 |
| Lurasidone |  |  |  |  |  |  |
| Absent | 70,467 | 100 | 56,376 | 100 | 14,091 | 99.99 |
| Present | 3 | 0 | 1 | 0 | 2 | 0.01 |
| Midazolam |  |  |  |  |  |  |
| Absent | 65,624 | 93.12 | 52,484 | 93.09 | 13,140 | 93.24 |
| Present | 4,846 | 6.88 | 3,893 | 6.91 | 953 | 6.76 |
| Quetiapine |  |  |  |  |  |  |
| Absent | 67,550 | 95.86 | 54,029 | 95.84 | 13,521 | 95.94 |
| Present | 2,920 | 4.14 | 2,348 | 4.16 | 572 | 4.06 |
| Saquinavir |  |  |  |  |  |  |
| Absent | 70,467 | 100 | 56,374 | 99.99 | 14,093 | 100 |
| Present | 3 | 0 | 3 | 0.01 | 0 | 0 |
| Sildenafil |  |  |  |  |  |  |
| Absent | 70,196 | 99.61 | 56,164 | 99.62 | 14,032 | 99.57 |
| Present | 274 | 0.39 | 213 | 0.38 | 61 | 0.43 |
| Sirolimus |  |  |  |  |  |  |
| Absent | 70,390 | 99.89 | 56,311 | 99.88 | 14,079 | 99.9 |
| Present | 80 | 0.11 | 66 | 0.12 | 14 | 0.1 |
| Tacrolimus |  |  |  |  |  |  |
| Absent | 69,858 | 99.13 | 55,912 | 99.18 | 13,946 | 98.96 |
| Present | 612 | 0.87 | 465 | 0.82 | 147 | 1.04 |
| Ticagrelor |  |  |  |  |  |  |
| Absent | 69,813 | 99.07 | 55,862 | 99.09 | 13,951 | 98.99 |
| Present | 657 | 0.93 | 515 | 0.91 | 142 | 1.01 |
| Tolvaptan |  |  |  |  |  |  |
| Absent | 70,388 | 99.88 | 56,307 | 99.88 | 14,081 | 99.91 |
| Present | 82 | 0.12 | 70 | 0.12 | 12 | 0.09 |
| Vardenafil |  |  |  |  |  |  |
| Absent | 70,445 | 99.96 | 56,360 | 99.97 | 14,085 | 99.94 |
| Present | 25 | 0.04 | 17 | 0.03 | 8 | 0.06 |
| **CYP3A4 weak inhibitors** | | | | |  |  |
| Cilostazol |  |  |  |  |  |  |
| Absent | 68,338 | 96.97 | 54,657 | 96.95 | 13,681 | 97.08 |
| Present | 2,132 | 3.03 | 1,720 | 3.05 | 412 | 2.92 |
| Fosaprepitant |  |  |  |  |  |  |
| Absent | 70,469 | 100 | 56,376 | 100 | 14,093 | 100 |
| Present | 1 | 0 | 1 | 0 | 0 | 0 |
| Ranitidine |  |  |  |  |  |  |
| Absent | 67,325 | 95.54 | 53,858 | 95.53 | 13,467 | 95.56 |
| Present | 3,145 | 4.46 | 2,519 | 4.47 | 626 | 4.44 |
| **CYP3A4 moderate inhibitors** | | | | | | |
| Cimetidine |  |  |  |  |  |  |
| Absent | 70,447 | 99.97 | 56,360 | 99.97 | 14,087 | 99.96 |
| Present | 23 | 0.03 | 17 | 0.03 | 6 | 0.04 |
| Ciprofloxacin |  |  |  |  |  |  |
| Absent | 65,625 | 93.12 | 52,519 | 93.16 | 13,106 | 93.00 |
| Present | 4,845 | 6.88 | 3,858 | 6.84 | 987 | 7.00 |
| Crizotinib |  |  |  |  |  |  |
| Absent | 70,463 | 99.99 | 56,371 | 99.99 | 14,092 | 99.99 |
| Present | 7 | 0.01 | 6 | 0.01 | 1 | 0.01 |
| Cyclosporine |  |  |  |  |  |  |
| Absent | 69,729 | 98.95 | 55,799 | 98.97 | 13,930 | 98.84 |
| Present | 741 | 1.05 | 578 | 1.03 | 163 | 1.16 |
| Erythromycin |  |  |  |  |  |  |
| Absent | 70,463 | 99.99 | 56,373 | 99.99 | 14,090 | 99.98 |
| Present | 7 | 0.01 | 4 | 0.01 | 3 | 0.02 |
| Fluvoxamine |  |  |  |  |  |  |
| Absent | 70,442 | 99.96 | 56,353 | 99.96 | 14,089 | 99.97 |
| Present | 28 | 0.04 | 24 | 0.04 | 4 | 0.03 |
| Imatinib |  |  |  |  |  |  |
| Absent | 70,409 | 99.91 | 56,323 | 99.9 | 14,086 | 99.95 |
| Present | 61 | 0.09 | 54 | 0.1 | 7 | 0.05 |
| Verapamil |  |  |  |  |  |  |
| Absent | 69,982 | 99.31 | 55,989 | 99.31 | 13,993 | 99.29 |
| Present | 488 | 0.69 | 388 | 0.69 | 100 | 0.71 |
| **CYP3A4 strong inhibitors** | | | | | | |
| Clarithromycin |  |  |  |  |  |  |
| Absent | 69,148 | 98.12 | 55,315 | 98.12 | 13,833 | 98.16 |
| Present | 1,322 | 1.88 | 1,062 | 1.88 | 260 | 1.84 |
| Cobicistat |  |  |  |  |  |  |
| Absent | 70,466 | 99.99 | 56,374 | 99.99 | 14,092 | 99.99 |
| Present | 4 | 0.01 | 3 | 0.01 | 1 | 0.01 |
| Diltiazem |  |  |  |  |  |  |
| Absent | 68,146 | 96.70 | 54,520 | 96.71 | 13,626 | 96.69 |
| Present | 2,324 | 3.30 | 1,857 | 3.29 | 467 | 3.31 |
| Itraconazole |  |  |  |  |  |  |
| Absent | 70,233 | 99.66 | 56,188 | 99.66 | 14,045 | 99.66 |
| Present | 237 | 0.34 | 189 | 0.34 | 48 | 0.34 |
| Ketoconazole |  |  |  |  |  |  |
| Absent | 70,317 | 99.78 | 56,258 | 99.79 | 14,059 | 99.76 |
| Present | 153 | 0.22 | 119 | 0.21 | 34 | 0.24 |
| Posaconazole |  |  |  |  |  |  |
| Absent | 70,459 | 99.98 | 56,369 | 99.99 | 14,090 | 99.98 |
| Present | 11 | 0.02 | 8 | 0.01 | 3 | 0.02 |
| Ritonavir |  |  |  |  |  |  |
| Absent | 70,406 | 99.91 | 56,320 | 99.90 | 14,086 | 99.95 |
| Present | 64 | 0.09 | 57 | 0.10 | 7 | 0.05 |
| Voriconazole |  |  |  |  |  |  |
| Absent | 70,422 | 99.93 | 56,337 | 99.93 | 14,085 | 99.94 |
| Present | 48 | 0.07 | 40 | 0.07 | 8 | 0.06 |
| **Glucuronidation** | | | | | | |
| **Glucuronidation substrates** | | | | | | |
| Entacapone |  |  |  |  |  |  |
| Absent | 70,204 | 99.62 | 56,154 | 99.6 | 14,050 | 99.69 |
| Present | 266 | 0.38 | 223 | 0.4 | 43 | 0.31 |
| Gemfibrozil |  |  |  |  |  |  |
| Absent | 69,054 | 97.99 | 55,233 | 97.97 | 13,821 | 98.07 |
| Present | 1,416 | 2.01 | 1,144 | 2.03 | 272 | 1.93 |
| Indomethacin |  |  |  |  |  |  |
| Absent | 70,350 | 99.83 | 56,281 | 99.83 | 14,069 | 99.83 |
| Present | 120 | 0.17 | 96 | 0.17 | 24 | 0.17 |
| lamotrigine |  |  |  |  |  |  |
| Absent | 70,374 | 99.86 | 56,298 | 99.86 | 14,076 | 99.88 |
| Present | 96 | 0.14 | 79 | 0.14 | 17 | 0.12 |
| Telmisartan |  |  |  |  |  |  |
| Absent | 69,420 | 98.51 | 55,563 | 98.56 | 13,857 | 98.33 |
| Present | 1,050 | 1.49 | 814 | 1.44 | 236 | 1.67 |
| Zidovudine |  |  |  |  |  |  |
| Absent | 70,414 | 99.92 | 56,332 | 99.92 | 14,082 | 99.92 |
| Present | 56 | 0.08 | 45 | 0.08 | 11 | 0.08 |
| **Glucuronidation inhibitors** | | | | | | |
| Amitriptyline |  |  |  |  |  |  |
| Absent | 68,268 | 96.88 | 54,608 | 96.86 | 13,660 | 96.93 |
| Present | 2,202 | 3.12 | 1,769 | 3.14 | 433 | 3.07 |
| Chlorpromazine |  |  |  |  |  |  |
| Absent | 70,397 | 99.9 | 56,321 | 99.9 | 14,076 | 99.88 |
| Present | 73 | 0.1 | 56 | 0.1 | 17 | 0.12 |
| Clomipramine |  |  |  |  |  |  |
| Absent | 70,452 | 99.97 | 56,363 | 99.98 | 14,089 | 99.97 |
| Present | 18 | 0.03 | 14 | 0.02 | 4 | 0.03 |
| Clonazepam |  |  |  |  |  |  |
| Absent | 67,044 | 95.14 | 53,648 | 95.16 | 13,396 | 95.05 |
| Present | 3,426 | 4.86 | 2,729 | 4.84 | 697 | 4.95 |
| Diazepam |  |  |  |  |  |  |
| Absent | 68,060 | 96.58 | 54,436 | 96.56 | 13,624 | 96.67 |
| Present | 2,410 | 3.42 | 1,941 | 3.44 | 469 | 3.33 |
| Ethinylestradiol |  |  |  |  |  |  |
| Absent | 70,402 | 99.90 | 56,327 | 99.91 | 14,075 | 99.87 |
| Present | 68 | 0.10 | 50 | 0.09 | 18 | 0.13 |
| Flunitrazepam |  |  |  |  |  |  |
| Absent | 70,256 | 99.70 | 56,208 | 99.70 | 14,048 | 99.68 |
| Present | 214 | 0.30 | 169 | 0.30 | 45 | 0.32 |
| Ibuprofen |  |  |  |  |  |  |
| Absent | 69,416 | 98.50 | 55,527 | 98.49 | 13,889 | 98.55 |
| Present | 1,054 | 1.50 | 850 | 1.51 | 204 | 1.45 |
| Imipramine |  |  |  |  |  |  |
| Absent | 70,310 | 99.77 | 56,247 | 99.77 | 14,063 | 99.79 |
| Present | 160 | 0.23 | 130 | 0.23 | 30 | 0.21 |
| Lorazepam |  |  |  |  |  |  |
| Absent | 56,993 | 80.88 | 45,621 | 80.92 | 11,372 | 80.69 |
| Present | 13,477 | 19.12 | 10,756 | 19.08 | 2,721 | 19.31 |
| Naproxen |  |  |  |  |  |  |
| Absent | 68,202 | 96.78 | 54,535 | 96.73 | 13,667 | 96.98 |
| Present | 2,268 | 3.22 | 1,842 | 3.27 | 426 | 3.02 |
| Nitrazepam |  |  |  |  |  |  |
| Absent | 70,469 | 100 | 56,376 | 100 | 14,093 | 100 |
| Present | 1 | 0 | 1 | 0 | 0 | 0 |
| Probenecid |  |  |  |  |  |  |
| Absent | 70,365 | 99.85 | 56,288 | 99.84 | 14,077 | 99.89 |
| Present | 105 | 0.15 | 89 | 0.16 | 16 | 0.11 |
| Propranolol |  |  |  |  |  |  |
| Absent | 68,949 | 97.84 | 55,184 | 97.88 | 13,765 | 97.67 |
| Present | 1,521 | 2.16 | 1,193 | 2.12 | 328 | 2.33 |
| Sertraline |  |  |  |  |  |  |
| Absent | 67,868 | 96.31 | 54,288 | 96.29 | 13,580 | 96.36 |
| Present | 2,602 | 3.69 | 2,089 | 3.71 | 513 | 3.64 |
| **P-glycoprotein** | | | | | | |
| **P-glycoprotein substrates** | | | | | | |
| Dabigatran |  |  |  |  |  |  |
| Absent | 69,947 | 99.26 | 55,967 | 99.27 | 13,980 | 99.2 |
| Present | 523 | 0.74 | 410 | 0.73 | 113 | 0.8 |
| Digoxin |  |  |  |  |  |  |
| Absent | 69,212 | 98.21 | 55,353 | 98.18 | 13,859 | 98.34 |
| Present | 1,258 | 1.79 | 1,024 | 1.82 | 234 | 1.66 |
| Fexofenadine |  |  |  |  |  |  |
| Absent | 66,893 | 94.92 | 53,545 | 94.98 | 13,348 | 94.71 |
| Present | 3,577 | 5.08 | 2,832 | 5.02 | 745 | 5.29 |
| **P-glycoprotein inhibitors** | | | | | | |
| Carvedilol |  |  |  |  |  |  |
| Absent | 64,604 | 91.68 | 51,648 | 91.61 | 12,956 | 91.93 |
| Present | 5,866 | 8.32 | 4,729 | 8.39 | 1,137 | 8.07 |
| Lapatinib |  |  |  |  |  |  |
| Absent | 70,467 | 100 | 56,374 | 99.99 | 14,093 | 100 |
| Present | 3 | 0 | 3 | 0.01 | 0 | 0 |
| Ranolazine |  |  |  |  |  |  |
| Absent | 70,452 | 99.97 | 56,361 | 99.97 | 14,091 | 99.99 |
| Present | 18 | 0.03 | 16 | 0.03 | 2 | 0.01 |
| **Other drugs induced RM** | | | | | | |
| **Other lipid-lowering agents** | | | | | | |
| Bezafibrate |  |  |  |  |  |  |
| Absent | 70,439 | 99.96 | 56,350 | 99.95 | 14,089 | 99.97 |
| Present | 31 | 0.04 | 27 | 0.05 | 4 | 0.03 |
| Ezetimibe |  |  |  |  |  |  |
| Absent | 63,506 | 90.12 | 50,814 | 90.13 | 12,692 | 90.06 |
| Present | 6,964 | 9.88 | 5,563 | 9.87 | 1,401 | 9.94 |
| **Psychiatric agents** | | | | | | |
| Bromazepam |  |  |  |  |  |  |
| Absent | 70,387 | 99.88 | 56,309 | 99.88 | 14,078 | 99.89 |
| Present | 83 | 0.12 | 68 | 0.12 | 15 | 0.11 |
| Chlordiazepoxide |  |  |  |  |  |  |
| Absent | 70,069 | 99.43 | 56,066 | 99.45 | 14,003 | 99.36 |
| Present | 401 | 0.57 | 311 | 0.55 | 90 | 0.64 |
| Clobazam |  |  |  |  |  |  |
| Absent | 70,336 | 99.81 | 56,277 | 99.82 | 14,059 | 99.76 |
| Present | 134 | 0.19 | 100 | 0.18 | 34 | 0.24 |
| Clorazepate |  |  |  |  |  |  |
| Absent | 69,904 | 99.2 | 55,931 | 99.21 | 13,973 | 99.15 |
| Present | 566 | 0.8 | 446 | 0.79 | 120 | 0.85 |
| Doxepin |  |  |  |  |  |  |
| Absent | 70,260 | 99.7 | 56,207 | 99.7 | 14,053 | 99.72 |
| Present | 210 | 0.3 | 170 | 0.3 | 40 | 0.28 |
| Fluoxetine |  |  |  |  |  |  |
| Absent | 69,742 | 98.97 | 55,780 | 98.94 | 13,962 | 99.07 |
| Present | 728 | 1.03 | 597 | 1.06 | 131 | 0.93 |
| Fluphenazine |  |  |  |  |  |  |
| Absent | 70,455 | 99.98 | 56,366 | 99.98 | 14,089 | 99.97 |
| Present | 15 | 0.02 | 11 | 0.02 | 4 | 0.03 |
| Haloperidol |  |  |  |  |  |  |
| Absent | 69,581 | 98.74 | 55,668 | 98.74 | 13,913 | 98.72 |
| Present | 889 | 1.26 | 709 | 1.26 | 180 | 1.28 |
| Lithium |  |  |  |  |  |  |
| Absent | 70,445 | 99.96 | 56,356 | 99.96 | 14,089 | 99.97 |
| Present | 25 | 0.04 | 21 | 0.04 | 4 | 0.03 |
| Perphenazine |  |  |  |  |  |  |
| Absent | 70,390 | 99.89 | 56,313 | 99.89 | 14,077 | 99.89 |
| Present | 80 | 0.11 | 64 | 0.11 | 16 | 0.11 |
| Phenobarbital |  |  |  |  |  |  |
| Absent | 70,421 | 99.93 | 56,343 | 99.94 | 14,078 | 99.89 |
| Present | 49 | 0.07 | 34 | 0.06 | 15 | 0.11 |
| Trifluoperazine |  |  |  |  |  |  |
| Absent | 70,466 | 99.99 | 56,373 | 99.99 | 14,093 | 100 |
| Present | 4 | 0.01 | 4 | 0.01 | 0 | 0 |
| Venlafaxine |  |  |  |  |  |  |
| Absent | 70,263 | 99.71 | 56,218 | 99.72 | 14,045 | 99.66 |
| Present | 207 | 0.29 | 159 | 0.28 | 48 | 0.34 |
| **Others** | | | | | | |
| Amphotericin B |  |  |  |  |  |  |
| Absent | 70,403 | 99.9 | 56,319 | 99.9 | 14,084 | 99.94 |
| Present | 67 | 0.1 | 58 | 0.1 | 9 | 0.06 |
| Azathioprine |  |  |  |  |  |  |
| Absent | 69,980 | 99.3 | 55,991 | 99.32 | 13,989 | 99.26 |
| Present | 490 | 0.7 | 386 | 0.68 | 104 | 0.74 |
| Diphenhydramine |  |  |  |  |  |  |
| Absent | 69,942 | 99.25 | 55,961 | 99.26 | 13,981 | 99.21 |
| Present | 528 | 0.75 | 416 | 0.74 | 112 | 0.79 |
| Hydrochlorothiazide |  |  |  |  |  |  |
| Absent | 64,427 | 91.42 | 51,556 | 91.45 | 12,871 | 91.33 |
| Present | 6,043 | 8.58 | 4,821 | 8.55 | 1,222 | 8.67 |
| Penicillamine |  |  |  |  |  |  |
| Absent | 70,469 | 100 | 56,376 | 100 | 14,093 | 100 |
| Present | 1 | 0 | 1 | 0 | 0 | 0 |
| Pentamidine |  |  |  |  |  |  |
| Absent | 70,462 | 99.99 | 56,369 | 99.99 | 14,093 | 100 |
| Present | 8 | 0.01 | 8 | 0.01 | 0 | 0 |
| Propofol |  |  |  |  |  |  |
| Absent | 70,391 | 99.89 | 56,316 | 99.89 | 14,075 | 99.87 |
| Present | 79 | 0.11 | 61 | 0.11 | 18 | 0.13 |
| Salicylates |  |  |  |  |  |  |
| Absent | 44,900 | 63.72 | 35,909 | 63.69 | 8,991 | 63.8 |
| Present | 25,570 | 36.28 | 20,468 | 36.31 | 5,102 | 36.2 |
| Succinylcholine |  |  |  |  |  |  |
| Absent | 70,294 | 99.75 | 56,234 | 99.75 | 14,060 | 99.77 |
| Present | 176 | 0.25 | 143 | 0.25 | 33 | 0.23 |
| Terbutaline |  |  |  |  |  |  |
| Absent | 70,451 | 99.97 | 56,361 | 99.97 | 14,090 | 99.98 |
| Present | 19 | 0.03 | 16 | 0.03 | 3 | 0.02 |
| Theophylline |  |  |  |  |  |  |
| Absent | 69,286 | 98.32 | 55,429 | 98.32 | 13,857 | 98.33 |
| Present | 1,184 | 1.68 | 948 | 1.68 | 236 | 1.67 |
| **Laboratory findings** | | | | | | |
| AST, median(range) | 28.00  (2.00, 18749.00) | | 28.00  (2.00, 18749.00) | | 28.00  (2.00, 5983.00) | |
| Ca, mean(sd) | 9.05 (0.67) | | 9.05 (0.67) | | 9.05 (0.67) | |
| eGFR, median(range) | 70.90  (0.20, 270.20) | | 70.90  (0.20, 239.80) | | 70.60  (1.40, 270.20) | |
| K, mean(sd) | 3.98 (0.47) | | 3.98 (0.47) | | 3.98 (0.47) | |
| LDL, median(range) | 112.00  (16.00, 784.00) | | 111.00  (16.00, 784.00) | | 112.00  (18.00, 542.00) | |
| Na, mean(sd) | 139.62 (3.14) | | 139.62 (3.14) | | 139.6 (3.15) | |
| PO_4_, mean(sd) | 3.42 (0.67) | | 3.42 (0.67) | | 3.42 (0.68) | |
| TG, median(range) | 127.00  (16.00, 9054.00) | | 127.00  (16.00, 9054.00) | | 127.00  (19.00, 2674.00) | |

**Table A.8 Distribution of imputed features**

| **Features** | **Train (N = 56,377)** | | **Test (N = 14,093)** | |
| --- | --- | --- | --- | --- |
|  | **Complete data** | **Imputed data** | **Complete data** | **Imputed data** |
| **AST** |  |  |  |  |
| Missing, N(%) | 8 (0.01%) |  | 1 (0.01%) |  |
| Mean (SD) | 49.09 (285.31) | 49.09 (285.29) | 45.10 (162.17) | 45.10 (162.16) |
| Median | 28.00 (2.00, 18749) | 28.00 (2.00, 18749) | 28.00 (2, 5983) | 28.00 (2, 5983) |
| **Ca** |  |  |  |  |
| Missing, N(%) | 24 (0.04%) |  | 4 (0.03%) |  |
| Mean | 9.05 (0.67) | 9.05 (0.67) | 9.05 (0.67) | 9.05 (0.67) |
| Median | 9.10 (3.00, 13.60) | 9.10 (3.00, 13.60) | 9.10 (4.40, 12.10) | 9.10 (4.40, 12.10) |
| **eGFR^*^** |  |  |  |  |
| Missing, N(%) | 2 (0.004%) |  | 1 (0.01%) |  |
| Mean | 65.96 (27.79) | 65.96 (27.79) | 65.93 (27.86) | 65.93 (27.86) |
| Median | 70.90 (0.20, 239.80) | 70.90 (0.20, 239.80) | 70.60 (1.40, 270.20) | 70.60 (1.40, 270.20) |
| **K** |  |  |  |  |
| Missing, N(%) | 9 (0.02%) |  | 1 (0.01%) |  |
| Mean | 3.98 (0.47) | 3.98 (0.47) | 3.98 (0.47) | 3.98 (0.47) |
| Median | 4.02 (1.22, 6.35) | 4.02 (1.22, 6.35) | 4.02 (1.29, 7.52) | 4.02 (1.29, 7.52) |
| **LDL** |  |  |  |  |
| Missing, N(%) | 41 (0.07%) |  | 8 (0.06%) |  |
| Mean | 117.73 (40.70) | 117.72 (40.68) | 117.96 (40.46) | 117.96 (40.45) |
| Median | 111.00 (16.00, 784.00) | 112.00 (16.00, 784.00) | 112.00 (18.00, 542.00) | 112.00 (18.00, 542.00) |
| **Na** |  |  |  |  |
| Missing, N(%) | 10 (0.02%) |  | 1 (0.01%) |  |
| Mean | 139.62 (3.14) | 139.62 (3.14) | 139.60 (3.15) | 139.60 (3.15) |
| Median | 140.00 (111.00, 174.00) | 140.00 (111.00, 174.00) | 140.00 (111.00, 160.00) | 140.00 (111.00, 160.00) |
| **PO_4_** |  |  |  |  |
| Missing, N(%) | 24 (0.04%) |  | 4 (0.03%) |  |
| Mean | 3.42 (0.67) | 3.42 (0.67) | 3.42 (0.68) | 3.42 (0.68) |
| Median | 3.40 (0.20, 13.50) | 3.40 (0.20, 13.50) | 3.50 (0.40, 14.20) | 3.50 (0.40, 14.20) |
| **TG** |  |  |  |  |
| Missing, N(%) | 43 (0.08%) |  | 6 (0.04%) |  |
| Mean | 149.07 (111.46) | 149.06 (111.41) | 147.90 (92.97) | 147.90 (92.95) |
| Median | 127.00 (16.00, 9054.00) | 127.00 (16.00, 9054.00) | 127.00 (19.00, 2674.00) | 127.00 (19.00, 2674.00) |

*Serum creatinine was imputed, then eGFR was calculated afterward

These features were imputed based on following equations:

$$Scr=b_{0}+b_{1}age+b_{2}sex+b_{3}conmorbid+b_{4}statin+\ldots{+b}_{114}\mathrm{Theophylline}$$

$$AST=b_{0}+b_{1}age+b_{2}sex+b_{3}conmorbid+b_{4}statin+\ldots{+b}_{114}Theophylline+b_{115}Scr$$

$$Na=b_{0}+b_{1}age+b_{2}sex+b_{3}conmorbid+b_{4}statin+\ldots{+b}_{114}Theophylline+b_{115}Scr+b_{116}AST$$

$$K=b_{0}+b_{1}age+b_{2}sex+b_{3}conmorbid+b_{4}statin+\ldots{+b}_{114}Theophylline+b_{115}Scr+b_{116}AST+b_{117}Na$$

$$Ca=b_{0}+b_{1}age+b_{2}sex+b_{3}conmorbid+b_{4}statin+\ldots{+b}_{114}Theophylline+b_{115}Scr+b_{116}AST+b_{117}Na+b_{118}K$$

$${PO}_{4}=b_{0}+b_{1}age+b_{2}sex+b_{3}conmorbid+b_{4}statin+\ldots{+b}_{114}Theophylline+b_{115}Scr+b_{116}AST+b_{117}Na+b_{118}K+b_{119}Ca$$

$$LDL=b_{0}+b_{1}age+b_{2}sex+b_{3}conmorbid+b_{4}statin+\ldots{+b}_{114}Theophylline+b_{115}Scr+b_{116}AST+b_{117}Na+b_{118}K+b_{119}Ca+b_{120}{PO}_{4}$$

$$TG=b_{0}+b_{1}age+b_{2}sex+b_{3}conmorbid+b_{4}statin+\ldots{+b}_{114}Theophylline+b_{115}Scr+b_{116}AST+b_{117}Na++b_{118}K+b_{119}Ca+b_{120}{PO}_{4}+b_{121}LDL$$

**Table A.9 Data distribution over the discretised features**

| **Discretised features** | **Train** | | **Test** | |
| --- | --- | --- | --- | --- |
|  | **N** | **%** | **N** | **%** |
| Age group |  |  |  |  |
| 0: <65 years old | 23,236 | 41.22 | 5,807 | 41.20 |
| 1: ≥65 years old | 33,141 | 58.78 | 8,286 | 58.80 |
| DDD group |  |  |  |  |
| 0: low-dose (≤1 DDD) | 42,845 | 76.00 | 10,712 | 76.01 |
| 1: high dose (>1 DDD) | 13,532 | 24.00 | 3,381 | 23.99 |
| AST group |  |  |  |  |
| 0: normal (≤30 U/L) | 33,837 | 60.02 | 8,421 | 59.75 |
| 1: abnormal (>30 U/L) | 22,540 | 39.98 | 5,672 | 40.25 |
| eGFR group |  |  |  |  |
| 0: eGFR ≥60 mL/min/1.73m^2^ | 35,677 | 63.28 | 8,906 | 63.19 |
| 1: eGFR <60 mL/min/1.73m^2^ | 20,700 | 36.72 | 5,187 | 36.81 |
| LDL group |  |  |  |  |
| 0: normal (≤125 mg/dL) | 36,122 | 64.07 | 8,981 | 63.73 |
| 1: abnormal (>125 mg/dL) | 20,255 | 35.93 | 5,112 | 36.27 |
| TG group |  |  |  |  |
| 0: normal (≤150 mg/dL) | 35,979 | 63.82 | 9,088 | 64.49 |
| 1: abnormal (>150 mg/dL) | 20,398 | 36.18 | 5,005 | 35.51 |
| Ca group |  |  |  |  |
| 0: normal (≥8.5 mg/dL) | 48,035 | 85.20 | 11,961 | 84.87 |
| 1: abnormal (<8.5 mg/dL) | 8,342 | 14.80 | 2,132 | 15.13 |
| K group |  |  |  |  |
| 0: normal (≥3.5 mmol/L) | 48,531 | 86.08 | 12,084 | 85.74 |
| 1: abnormal (<3.5 mmol/L) | 7,846 | 13.92 | 2,009 | 14.26 |
| Na group |  |  |  |  |
| 0: normal (135-145 mmol/L) | 52,597 | 93.30 | 13,120 | 93.10 |
| 1: abnormal (<135 or >145 mmol/L) | 3,780 | 6.70 | 973 | 6.90 |
| PO_4_ group |  |  |  |  |
| 0: normal (≥2.5 mg/dL) | 52,647 | 93.38 | 13,132 | 93.18 |
| 1: abnormal (<2.5 mg/dL) | 3,730 | 6.62 | 961 | 6.82 |

**Table A.10 Selected features based on the expert’s opinion and univariate analysis**

| **Included Features** | **Possible values** | **Univariate analysis** | |
| --- | --- | --- | --- |
|  |  | **χ^2^** | **p-value** |
| Statin groups | 0: low-dose hydrophilic  1: high-dose hydrophilic  2: low-dose lipophilic  3: high-dose lipophilic | 1.017^b^  11.824  17.108 | 0.771^a^  0.001*  <0.001* |
| Amiodarone | 0: absent, 1: present | 1.697^b^ | 0.231^a^ |
| Antifungals | 0: absent, 1: present | 4.050 ^b,c^ | 0.091* |
| Carvedilol | 0: absent, 1: present | 13.867 | <0.001* |
| Ciprofloxacin | 0: absent, 1: present | 0.012 | 0.914^a^ |
| Clarithromycin | 0: absent, 1: present | 3.307 ^b^ | 0.021* |
| Colchicine | 0: absent, 1: present | 3.048^b^ | 0.003* |
| Cyclosporine | 0: absent, 1: present | 3.589^b^ | 0.056* |
| Digoxin | 0: absent, 1: present | 1.319^b^ | 0.667 ^a^ |
| Diltiazem | 0: absent, 1: present | 1.469^b^ | 0.360 ^a^ |
| Ezetimibe | 0: absent, 1: present | 0.006 | 0.938 ^a^ |
| Gemfibrozil | 0: absent, 1: present | 0.581^b^ | 1.000 ^a^ |
| Ticagrelor | 0: absent, 1: present | 4.035^b^ | 0.042* |
| Age | 0: <65 years old  1: ≥65 years old | 0.479 | 0.489 ^a^ |
| HTN | 0: absent, 1: present | 12.445 | <0.001* |
| AST | 0: normal (≤30 U/L)  1: abnormal (>30 U/L) | 109.363 | <0.001* |
| eGFR | 0: eGFR ≥60 mL/min/1.73m^2^  1: eGFR <60 mL/min/1.73m^2^ | 33.779 | <0.001* |
| LDL | 0: normal (≤125 mg/dL)  1: abnormal (>125 mg/dL) | 12.733 | <0.001* |

^a^ Features were included based on expert’s opinions even though they were not statistically significant.

^b^ Fisher exact test was applied because there was an expected value less than 5 or more than 20 percent among the feature and outcome.

^c^ Antifungals were combination features of ketoconazole, itraconazole, and voriconazole.

* Significance at p-value ≤ 0.1

**Table A.11 The conditional probability of an outcome given features**

| **Features** | **Probability of outcome** |
| --- | --- |
| **Outcome (conditional probability**  **based on train dataset)** | 3.13% |
| **Statin groups** |  |
| 1. Low-dose hydrophilic | 6.35% (++) |
| 2. High-dose hydrophilic | 8.54% (++) |
| 3. Low-dose lipophilic | 2.35% (-) |
| 4. High-dose lipophilic | 3.36% (+) |
| **Medications** |  |
| Antifungals | 25.15% (+++*) |
| Ticagrelor | 24.94% (+++*) |
| Cyclosporine | 23.03% (+++*) |
| Digoxin | 20.87% (+++*) |
| Clarithromycin | 16.19% (+++*) |
| Amiodarone | 14.99% (+++*) |
| Gemfibrozil | 14.81% (+++*) |
| Diltiazem | 13.55% (+++*) |
| Colchicine | 12.32% (+++) |
| Ciprofloxacin | 9.01% (++) |
| Carvedilol | 8.76% (++) |
| Ezetimibe | 7.44% (++) |
| **Other covariables** |  |
| eGFR (<60 mL/min/1.73m^2^) | 5.64% (+) |
| LDL (>125 mg/dL) | 3.93% (+) |
| AST (>30 U/L) | 3.08% (-) |
| Age (>65 years old) | 2.69% (-) |
| Hypertension | 2.60% (-) |

+, ++, +++, +++* The probability of an outcome given feature had 1, 2, 3, and 4 times or more above higher, respectively, compared to the conditional probability of outcome obtained from the train dataset.

**Table A. 12 Conditional probabilities of the outcome (%) predicted from concurrent using low-dose hydrophilic statins with other 2 drugs**

| **Drugs** | **D01** | **D02** | **D03** | **D04** | **D05** | **D06** | **D07** | **D08** | **D09** | **D10** | **D11** |
| --- | --- | --- | --- | --- | --- | --- | --- | --- | --- | --- | --- |
| **D02** | 49.87 |  |  |  |  |  |  |  |  |  |  |
| **D03** | 41.74 | 49.96 |  |  |  |  |  |  |  |  |  |
| **D04** | 41.45 | 50.00 | 43.76 |  |  |  |  |  |  |  |  |
| **D05** | 47.13 | 48.30 | 47.54 | 43.62 |  |  |  |  |  |  |  |
| **D06** | 44.05 | 50.00 | 45.17 | 38.91 | 46.27 |  |  |  |  |  |  |
| **D07** | 49.98 | 50.00 | 48.71 | 50.00 | 50.00 | 50.00 |  |  |  |  |  |
| **D08** | 45.50 | 49.92 | 45.58 | 44.48 | 50.00 | 48.38 | 50.00 |  |  |  |  |
| **D09** | 42.50 | 50.00 | 44.37 | 45.21 | 45.01 | 47.68 | 44.11 | 48.45 |  |  |  |
| **D10** | 42.00 | 47.98 | 33.32 | 32.33 | 45.40 | 39.39 | 48.24 | 49.14 | 39.53 |  |  |
| **D11** | 50.00 | 48.98 | 49.48 | 47.24 | 50.00 | 48.40 | 50.00 | 50.00 | 50.00 | 38.62 |  |
| **D12** | 49.78 | 50.00 | 49.83 | 44.91 | 47.64 | 49.83 | 50.00 | 50.00 | 49.94 | 49.83 | 49.17 |

D01=Amiodarone, D02=Antifungals, D03=Carvedilol, D04=Ciprofloxacin, D05=Clarithromycin, D06=Colchicine, D07=Cyclosporine, D08=Digoxin, D09=Diltiazem, D10=Ezetimibe, D11=Gemfibrozil, D12=Ticagrelor

Highlight showed the highest probability of the outcome for each statin group.

**Table A.13 Conditional probabilities of the outcome (%) predicted from concurrent using high-dose hydrophilic statins with other 2 drugs**

| **Drugs** | **D01** | **D02** | **D03** | **D04** | **D05** | **D06** | **D07** | **D08** | **D09** | **D10** | **D11** |
| --- | --- | --- | --- | --- | --- | --- | --- | --- | --- | --- | --- |
| **D02** | 50.00 |  |  |  |  |  |  |  |  |  |  |
| **D03** | 44.24 | 50.00 |  |  |  |  |  |  |  |  |  |
| **D04** | 45.79 | 48.94 | 40.78 |  |  |  |  |  |  |  |  |
| **D05** | 49.89 | 50.00 | 46.20 | 44.75 |  |  |  |  |  |  |  |
| **D06** | 47.03 | 49.05 | 45.92 | 41.14 | 49.89 |  |  |  |  |  |  |
| **D07** | 50.00 | 50.00 | 49.84 | 48.38 | 50.00 | 49.99 |  |  |  |  |  |
| **D08** | 45.92 | 50.00 | 48.08 | 49.75 | 50.00 | 49.62 | 50.00 |  |  |  |  |
| **D09** | 48.63 | 50.00 | 45.24 | 45.40 | 49.15 | 49.46 | 48.42 | 49.84 |  |  |  |
| **D10** | 39.01 | 48.31 | 24.09 | 35.05 | 45.35 | 36.79 | 47.79 | 49.90 | 39.65 |  |  |
| **D11** | 50.00 | 47.75 | 42.26 | 47.50 | 50.00 | 49.54 | 50.00 | 49.99 | 45.89 | 32.21 |  |
| **D12** | 48.31 | 50.00 | 48.51 | 49.81 | 50.00 | 49.99 | 49.95 | 50.00 | 48.81 | 42.01 | 50.00 |

D01=Amiodarone, D02=Antifungals, D03=Carvedilol, D04=Ciprofloxacin, D05=Clarithromycin, D06=Colchicine, D07=Cyclosporine, D08=Digoxin, D09=Diltiazem, D10=Ezetimibe, D11=Gemfibrozil, D12=Ticagrelor

Highlight showed the highest probability of the outcome for each statin group.

**Table A.14 Conditional probabilities of the outcome (%) predicted from concurrent using low-dose lipophilic statins with other 2 drugs**

| **Drugs** | **D01** | **D02** | **D03** | **D04** | **D05** | **D06** | **D07** | **D08** | **D09** | **D10** | **D11** |
| --- | --- | --- | --- | --- | --- | --- | --- | --- | --- | --- | --- |
| **D02** | 44.57 |  |  |  |  |  |  |  |  |  |  |
| **D03** | 20.61 | 43.82 |  |  |  |  |  |  |  |  |  |
| **D04** | 30.76 | 35.56 | 20.03 |  |  |  |  |  |  |  |  |
| **D05** | 47.26 | 46.72 | 38.95 | 19.83 |  |  |  |  |  |  |  |
| **D06** | 37.52 | 44.97 | 22.29 | 25.11 | 36.23 |  |  |  |  |  |  |
| **D07** | 49.04 | 41.71 | 35.56 | 31.79 | 46.54 | 43.21 |  |  |  |  |  |
| **D08** | 24.14 | 48.44 | 25.47 | 35.12 | 49.71 | 39.91 | 49.98 |  |  |  |  |
| **D09** | 28.89 | 39.49 | 26.91 | 28.95 | 39.66 | 29.79 | 31.88 | 34.17 |  |  |  |
| **D10** | 30.23 | 36.28 | 15.76 | 17.99 | 28.80 | 23.44 | 39.21 | 43.71 | 24.73 |  |  |
| **D11** | 45.17 | 45.31 | 35.82 | 27.07 | 37.75 | 31.79 | 45.99 | 47.81 | 34.67 | 24.82 |  |
| **D12** | 48.34 | 49.89 | 34.07 | 46.61 | 46.74 | 43.94 | 48.43 | 49.68 | 45.33 | 40.29 | 47.38 |

D01=Amiodarone, D02=Antifungals, D03=Carvedilol, D04=Ciprofloxacin, D05=Clarithromycin, D06=Colchicine, D07=Cyclosporine, D08=Digoxin, D09=Diltiazem, D10=Ezetimibe, D11=Gemfibrozil, D12=Ticagrelor

Highlight showed the top 5 highest probability of the outcome for each statin group.

**Table A.15 Conditional probabilities of the outcome (%) predicted from concurrent using high-dose lipophilic statins with other 2 drugs**

| **Drugs** | **D01** | **D02** | **D03** | **D04** | **D05** | **D06** | **D07** | **D08** | **D09** | **D10** | **D11** |
| --- | --- | --- | --- | --- | --- | --- | --- | --- | --- | --- | --- |
| **D02** | 46.83 |  |  |  |  |  |  |  |  |  |  |
| **D03** | 23.31 | 42.51 |  |  |  |  |  |  |  |  |  |
| **D04** | 31.80 | 43.40 | 19.95 |  |  |  |  |  |  |  |  |
| **D05** | 46.82 | 48.71 | 41.16 | 37.71 |  |  |  |  |  |  |  |
| **D06** | 35.63 | 48.66 | 23.18 | 27.24 | 40.41 |  |  |  |  |  |  |
| **D07** | 47.36 | 47.75 | 41.65 | 44.80 | 49.62 | 48.86 |  |  |  |  |  |
| **D08** | 25.42 | 48.47 | 31.02 | 40.13 | 49.86 | 40.97 | 49.96 |  |  |  |  |
| **D09** | 26.46 | 48.48 | 24.61 | 35.68 | 48.58 | 37.47 | 43.38 | 41.33 |  |  |  |
| **D10** | 28.63 | 44.23 | 13.12 | 18.11 | 34.65 | 24.35 | 38.58 | 40.07 | 28.31 |  |  |
| **D11** | 49.58 | 49.89 | 37.99 | 36.39 | 47.48 | 46.29 | 47.52 | 49.95 | 45.72 | 26.29 |  |
| **D12** | 42.01 | 49.99 | 24.08 | 44.26 | 43.95 | 39.74 | 49.91 | 48.19 | 41.40 | 32.31 | 49.95 |

D01=Amiodarone, D02=Antifungals, D03=Carvedilol, D04=Ciprofloxacin, D05=Clarithromycin, D06=Colchicine, D07=Cyclosporine, D08=Digoxin, D09=Diltiazem, D10=Ezetimibe, D11=Gemfibrozil, D12=Ticagrelor

Highlight showed the top 5 highest probabilities of the outcome for each statin group.

**Table A.16 The highest predicted probability of an outcome based on a group of statin users**

| **Features** | **Scenario** | | | | |
| --- | --- | --- | --- | --- | --- |
|  | **1** | **2** | **3** | **4** | **5** |
| **Group of**  **statin users** | Unknown  (any groups) | Low-dose  hydrophilic | High-dose  hydrophilic | Low-dose  lipophilic | High-dose  lipophilic |
| **Co-medications** | Colchicine | Clarithromycin | Antifungals | Colchicine | Carvedilol |
|  | Cyclosporine | Colchicine | Ezetimibe | Cyclosporine | Ticagrelor |
|  | Diltiazem | Diltiazem |  | Diltiazem | Gemfibrozil |
|  | Ezetimibe |  |  | Ezetimibe |  |
| **Co-variables** |  |  |  |  |  |
| Age (>65 years old) | No | Yes | No | No | Yes |
| Hypertension | Yes | Yes | No | Yes | Yes |
| eGFR  (<60 mL/min/1.73m^2^) | Yes | Yes | Yes | Yes | Yes |
| LDL (>125 mg/dL) | No | No | No | No | No |
| AST (>30 U/L) | Yes | Yes | No | Yes | Yes |
| **Probability of outcome** | 75.75% | 79.27% | 64.92% | 80.54% | 79.46% |
