# Supplementary material for: Detection of statin-induced rhabdomyolysis and muscular related adverse events through data mining technique
Source: BMC Med Inform Decis Mak. 2022 Sep 5;22:233. doi: 10.1186/s12911-022-01978-4 (PMC9446837; doi:10.1186/s12911-022-01978-4)

**Appendix Figures**

Fig. A.1 Possible pattern of patient's timeline

Fig. A.2 Top 10 co-administration drugs based on original, train, and test dataset

**Fig. A.1 Possible pattern of patient's timeline**

**
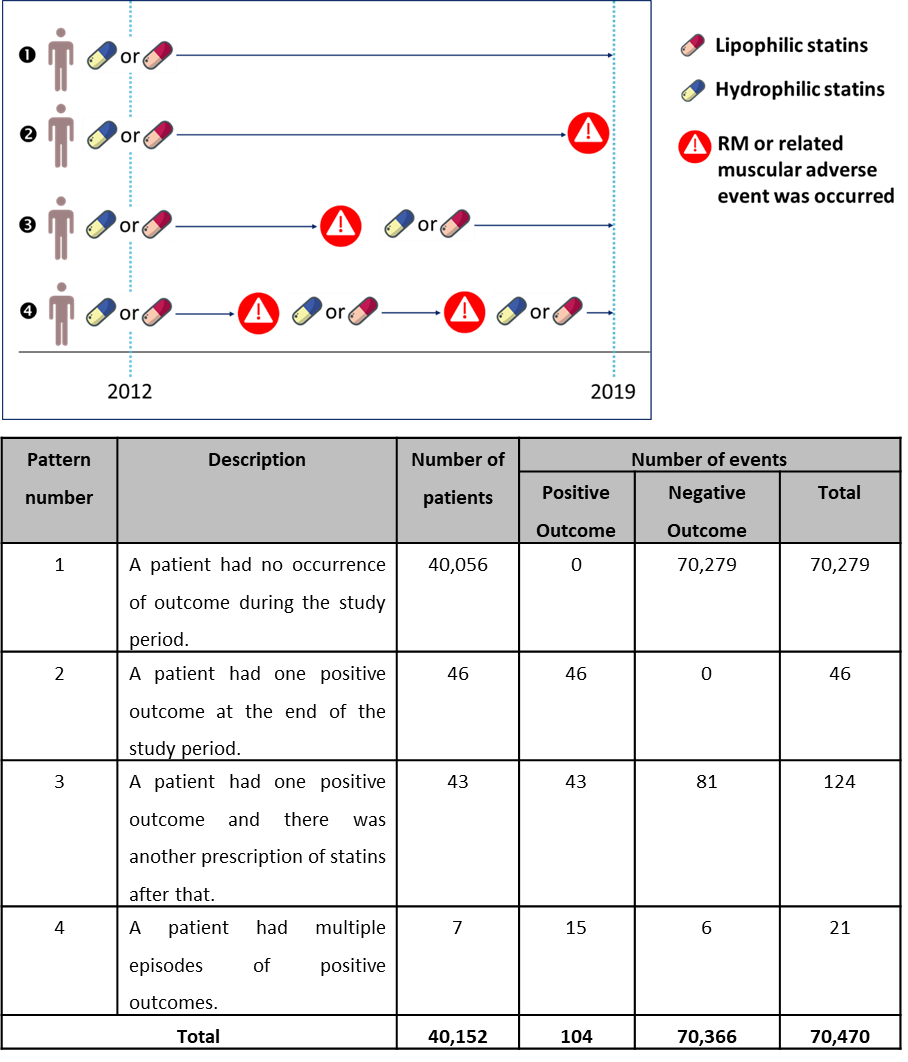
**

**Fig. A.2 Top 10 co-administration drugs based on original, train, and test dataset**


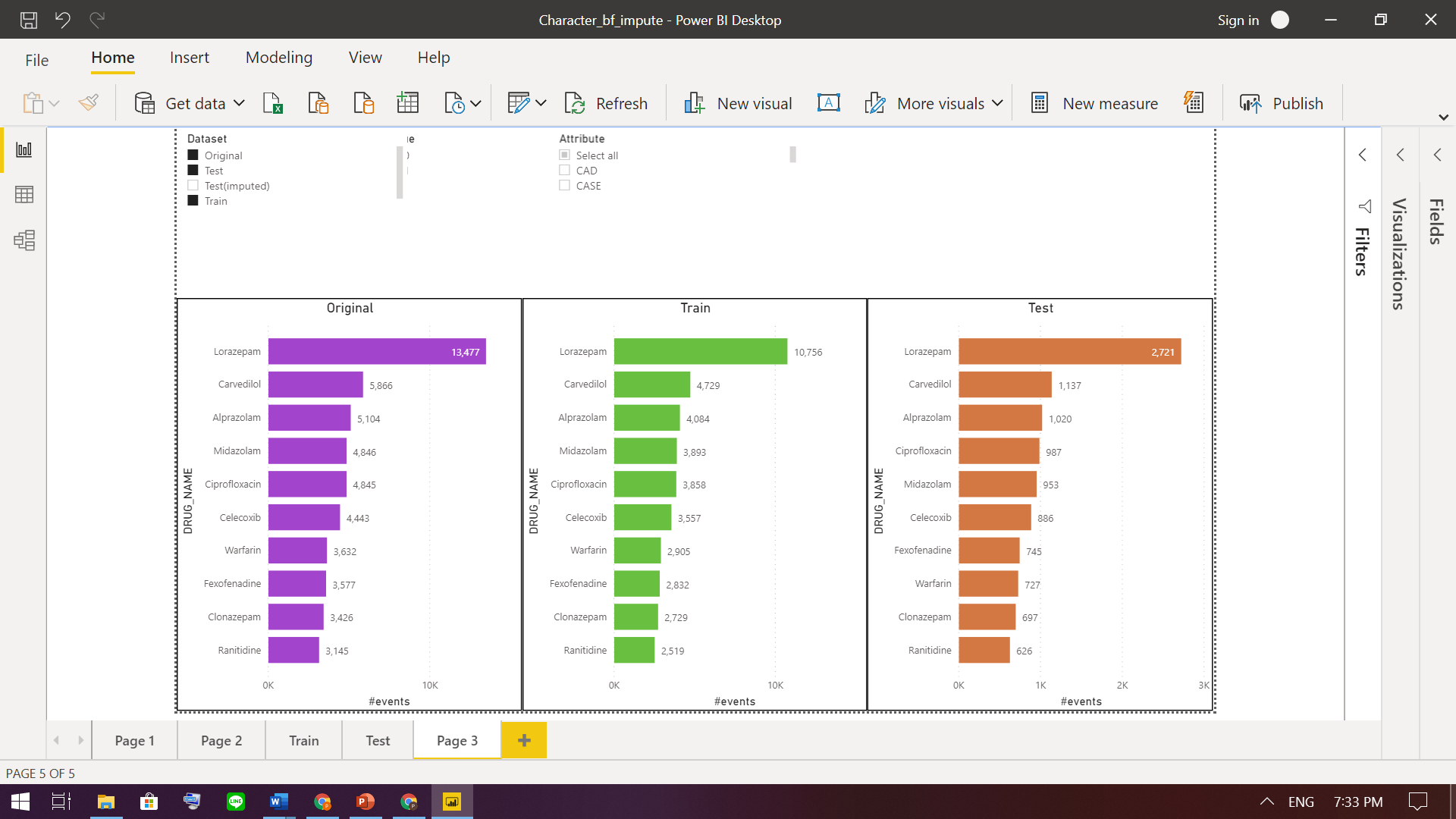

Supplement: Supplementary file 2 — Additional file 2. Fig. A1. [file 12911_2022_1978_MOESM2_ESM.docx]
